# Design, Synthesis, and Anti-Hepatocellular Carcinoma Evaluation of Sesquiterpene Lactone Epimers Trilobolide-6-*O*-isobutyrate Analogs

**DOI:** 10.3390/molecules29020393

**Published:** 2024-01-12

**Authors:** Xiuqiao Zhou, Guohui Yi, Yiming Qian, Xiaorong Yang, Guangying Chen, Yang Hui, Wenhao Chen

**Affiliations:** 1 Key Laboratory of Tropical Medicinal Resource Chemistry of Ministry of Education, College of Chemistry and Chemical Engineering, Hainan Normal University, Haikou 571158, China; m18708968677@163.com (X.Z.); 13485909409@163.com (Y.Q.); yangxr0902@163.com (X.Y.); chgying123@163.com (G.C.); 070066@hainnu.edu.cn (Y.H.); 2 Key Laboratory of Tropical Medicinal Plant Chemistry of Hainan Province, College of Chemistry and Chemical Engineering, Hainan Normal University, Haikou 571158, China; 3 Public Research Center, Hainan Medical University, Haikou 571199, China; guohuiyi6@hainmc.edu.cn

**Keywords:** sesquiterpene lactones, synthesis, anti-hepatocellular carcinoma evaluation

## Abstract

Hepatocellular carcinoma (HCC), one of the most common malignant cancers with a low 5-year survival rate, is the third leading cause of cancer-related deaths worldwide. The finding of novel agents and strategies for the treatment of HCC is an urgent need. Sesquiterpene lactones (SLs) have attracted extensive attention because of their potent antitumor activity. In this study, a new series of SL derivatives (**3**–**18**) were synthesized using epimers **1** and **2** as parent molecules, isolated from *Sphagneticola trilobata*, and evaluated for their anti-HCC activity. Furthermore, the structures of **4**, **6**, and **14** were confirmed by X-ray single-crystal diffraction analyses. The cytotoxic activities of **3**–**18** on two HCC cell lines, including HepG2 and Huh7, were evaluated using the CCK-8 assay. Among them, compound **10** exhibited the best activity against the HepG2 and Huh7 cell lines. Further studies showed that **10** induced cell apoptosis, arrested the cell cycle at the S phase, and induced the inhibition of cell proliferation and migration in HepG2 and Huh7. In addition, absorption, distribution, metabolism, and excretion (ADME) properties prediction showed that **10** may possess the properties to be a drug candidate. Thus, **10** may be a promising lead compound for the treatment of HCC.

## 1. Introduction

Cancer is a persistent unmet global challenge claiming millions of lives yearly and taking a tremendous toll on healthcare services and world economies [1]. According to the 2020 WHO report, liver cancer has the sixth highest incidence rate (4.7%) and the third highest mortality rate (8.3%) among the 36 types of cancer, making it one of the deadliest forms of cancer [2]. The most common primary liver cancer, hepatocellular carcinoma (HCC), is one of the most prevalent and lethal tumors [3]. As our understanding of HCC biology forges ahead, a number of first- and second-line chemotherapeutic drugs, as well as new targeted agents, immune checkpoint inhibitors (ICIs), and their combination therapies, are among the options available to patients [4]. Despite progress in this field, the effectiveness of most of them is severely limited by drug resistance, as well as invasion and metastasis of cancers [5,6]. The development of new effective anti-HCC drugs is therefore essential.

**Figure 1:**
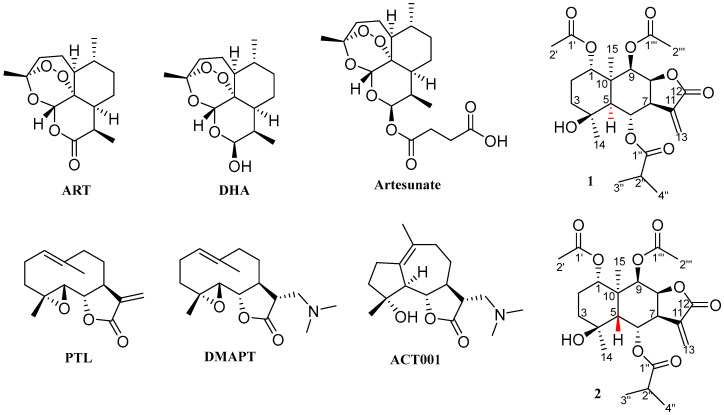
Structures of ART, DHA, Artesunate, PTL, DMAPT, and ACT001 **1** and **2**.

Natural sesquiterpene lactones (SLs) are a family of secondary metabolites derived almost exclusively from Asteraceae/Compositae plants [7]. These natural products have been a hot topic in the field of tumor drug research because of their complexity, diversity, novelty of structures, and uniqueness of function [8]. In recent years, SL-derived drugs such as derivatives of Artemisinin (ART) and Parthenolide (PTL) (Figure 1), have shown promise for the potential treatment of various types of cancer [9,10,11,12,13,14]. ART and its derivatives such as dihydroartemisinin (DHA) and artesunate could promote cancer cell apoptosis, induce cell cycle arrest and autophagy, and inhibit cancer cell invasion as well as migration, and they represent promising candidates for cancer therapy, supported by some clinical phase I/II trials [15,16,17,18]. PTL showed promising anti-cancer properties. Nevertheless, PTL has some disadvantages, such as poor oral bioavailability and poor water solubility [19,20]. DMAPT, a derivative of PTL, effectively increased the water solubility and oral bioavailability, and has advanced into a phase I clinical trial for the treatment of acute myeloid leukemia [21]. And the other PTL derivative, ACT001, has been clinically tested in Australia and China for the treatment of glioblastoma [22].

SLs’ cytotoxic activity has been described as mainly dependent on the *α*-methylene-*γ*-lactone group, which is prone to react with suitable nucleophiles [23,24,25]. Trilobolide-6-*O*-isobutyrate A and B (**1** and **2**), a pair of C-5 epimers of SLs with high oxygen content, were isolated from the flowers of *S. trilobata*, they also have an *α*-methylene-*γ*-lactone group. Subsequent investigations indicated that **2** could strongly inhibit the proliferation of HCC through inhibition of the IL-6/STAT3 signaling pathway [26]. And a new series of SL derivatives were designed and synthesized using **1** and **2** as the parent molecules. Unfortunately, all of the compounds did not show significant in vitro antiproliferative activity against the tested human cancer cell lines [27]. 

Based on our ongoing pursuit of the investigation of **1** and **2** to further enhance their antitumor profile, a series of **1** and **2** derivatives (**3**–**18**) were further designed and synthesized in this work. All the compounds were evaluated for their potential inhibition of HCC cell lines. We further investigated the effects of the most active compound **10** on HepG2 and Huh7 cells, and computational absorption, distribution, metabolism, and excretion (ADME) estimation studies were performed.

## 2. Results and Discussion

### 2.1. Synthesis

**Scheme 1:**
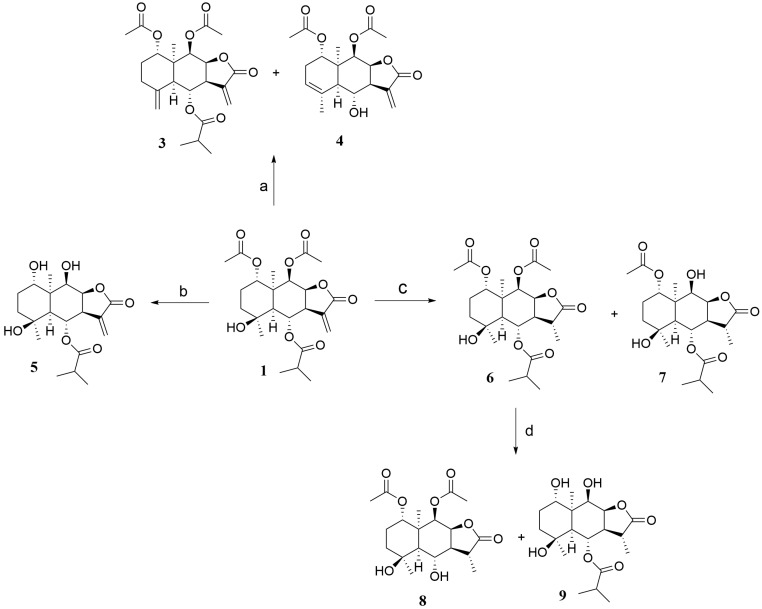
Synthesis route to obtain **1** derivatives. *Reagents and conditions*: (**a**) THF, HCl (2M, aq), 45 °C, 26 h; (**b**) KOH (aq), THF:H_2_O = 1:1, 0 °C, 2 h; (**c**) NaBH_4_, CH_3_CN, rt, 1 h; (**d**) KOH, MeOH, rt, 6 h.

**Scheme 2:**
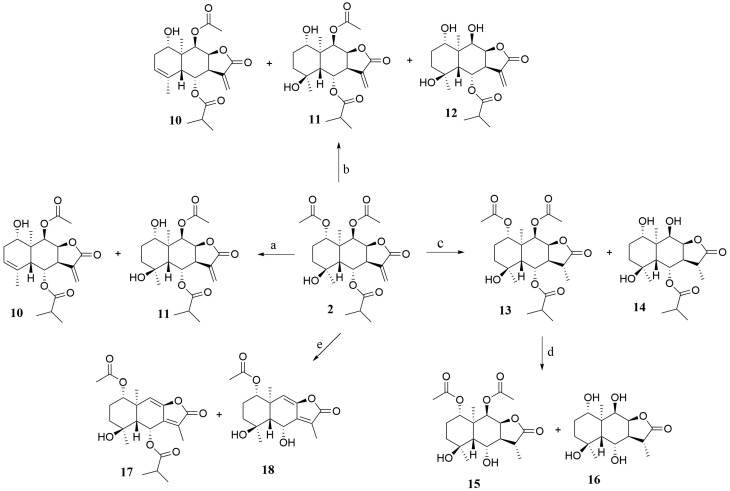
Synthesis route to obtain **2** derivatives. *Reagents and conditions*: (**a**) THF, HCl (2M, aq), 45 °C 9 h; (**b**) THF, H_2_SO_4_ (8M, aq), 45 °C, 10 h; (**c**) NaBH_4_, CH_3_CN, rt, 1 h; (**d**) KOH, MeOH, rt, 6 h; (**e**) DMSO, DBU, rt, 79 h.

Compounds **1** and **2** were isolated from the EtOH solutions of the dried flowers of *S. trilobata* by repeated column chromatography (CC) and these two compounds were further purified by recrystallization from EtOH following previously described methods [28]. In order to investigate whether the presence of ester groups and terminal double bonds in **1** and **2** has a positive effect on anti-hepatocellular carcinoma activities, we designed synthetic routes primarily considering the hydrolysis reaction. The acetyl groups at the C-1 and C-9 positions and the isobutyryl group at the C-6 position were removed, and two acetyl groups and one isobutyryl substituent on **1** and **2** were removed by selecting different acid and base systems to obtain hydrolysates with different degrees of ester removal. We used NaBH_4_ as a reductant to reduce the exocyclic double bond of C-11 (13) to the methyl group. Scheme 1 and Scheme 2 represent the synthetic pathways of all the compounds, respectively. 

First, **1** was dissolved in THF and hydrolyzed by HCl solution. The crude product was further purified by silica gel CC to obtain pure products **3** and **4** [29]. Then, **1** was dissolved in THF and H_2_O, the hydrolysis condition was changed to alkaline KOH solution, and the reaction was at 0 °C for 2 h to prepare the pure product **5**. Subsequently, the appropriate **1** and NaBH_4_ solids were dissolved in the CH_3_CN and stirred at 60 °C for 26.5 h to afford pure products **6** and **7** by HPLC [30]. In order to obtain different degrees of removal of the ester groups at the C-1, C-6, and C-9 positions and expose the hydroxyl groups, we switched to using KOH to provide alkaline conditions and MeOH as the solvent. The reaction mixture was stirred at room temperature for 6 h. Then, crude products were further purified by silica gel CC to afford pure products **8** and **9**. In the hydrolysis of **2**, we also optimized different acid-base systems under the conditions of HCl or H_2_SO_4_ solution, and prepared the products **10** and **12**. The hydrolysis derivatives **13**–**16** of **2** were also prepared according to the synthesis method of compounds **6**–**9**. Finally, **2** was dissolved in DMSO and reacted in the presence of DBU to obtain products **17** and **18** [31]. 

The structures of these derivatives were firmly established by combination with the physical and chemical properties, and 1D/2D NMR and HRMS methods. The Michael addition at the *α*-methylene-*γ*-lactone of SLs with amines has been found to be highly stereo-specific and yielded exclusively a single stereoisomer with the (R*)- configuration at the newly formed C-11 chiral carbon. This was attributed to the stereo-exclusive protonation of the enolate formed during the Michael addition [32]. In this research, firstly, the absolute configuration of the parent molecules **1** and **2** was determined by X-ray crystal analysis [26]. Secondly, in our previous work, the 11*R* stereochemistry of the SLs derivatives was confirmed by the NOE effect between H-11 and H-6 [27]. Finally, the X-ray crystal structures of **4**, **6**, and **14** (Figure S55) further confirmed the *R* configuration at C-11.

### 2.2. Biological Evaluation

#### 2.2.1. Cell Viability Assay and Structure–Activity Relationships (SARs)

The anti-HCC activity of 1, 2, and their derivatives was evaluated by the CCK-8 assay against human HCC cell lines HepG2 and Huh7, and compared with adriamycin as the working standard. As shown in Table 1, some derivatives showed comparatively significant IC_50_ values compared to 1 and 2. On the basis of the above activity data, a preliminary SARs was deduced. In the hydrolysates of 1 and 2, the cytotoxicity of compounds 3 and 10 was comparable or better than that of the parent molecules. It was suggested that the double bonds at the C-4, 14 or C-3, 4 positions were more favorable to enhancing the activity of HCC. Notably, the double bond between C-11 and C-13 was reduced to the methyl group in all the compounds, which were less potent than the parental compounds, further confirming SLs’ cytotoxic activity as being mainly dependent on the α-methylene-γ-lactone group [23,24,25]. Compound 10 demonstrated the best antiproliferative activity against HepG2 and Huh7 cells with IC_50_ values of 9.73 and 18.86 µM, respectively. Therefore, 10 was selected for subsequent antitumor experiments on HepG2 and Huh7 cell lines in vitro.

#### 2.2.2. Anti-Proliferative Effect of Compound **10** on Huh7 and HepG2 Cells

**Figure 2:**
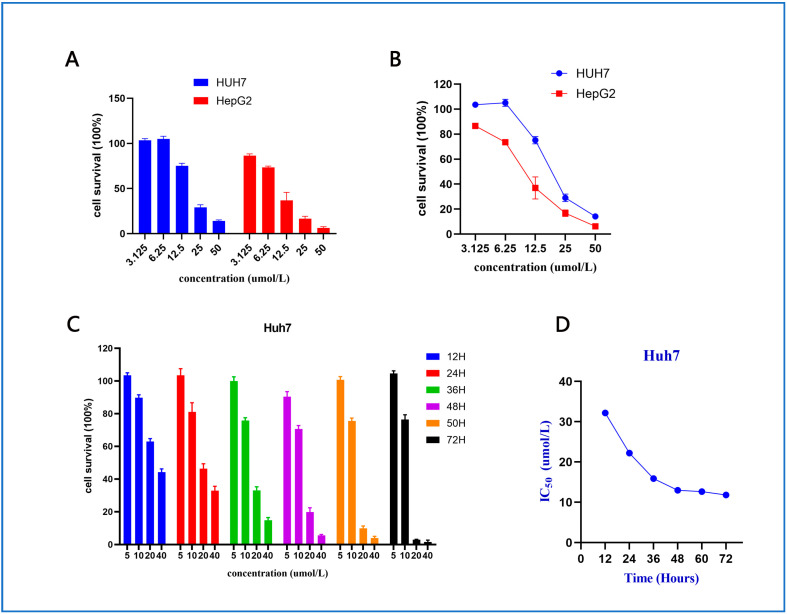
Cell viability of Huh7 and HepG2 cells. (**A**,**B**) Three cells were treated with indicated concentrations of compound **10** (3.125, 6.25, 12.5, 25, and 50 μM) for 24 h. (**C**,**D**) Cell viability of Huh7 cells treated with **10** at different concentrations for 12, 24, 26, 48, 60, and 72 h, respectively.

**Figure 3:**
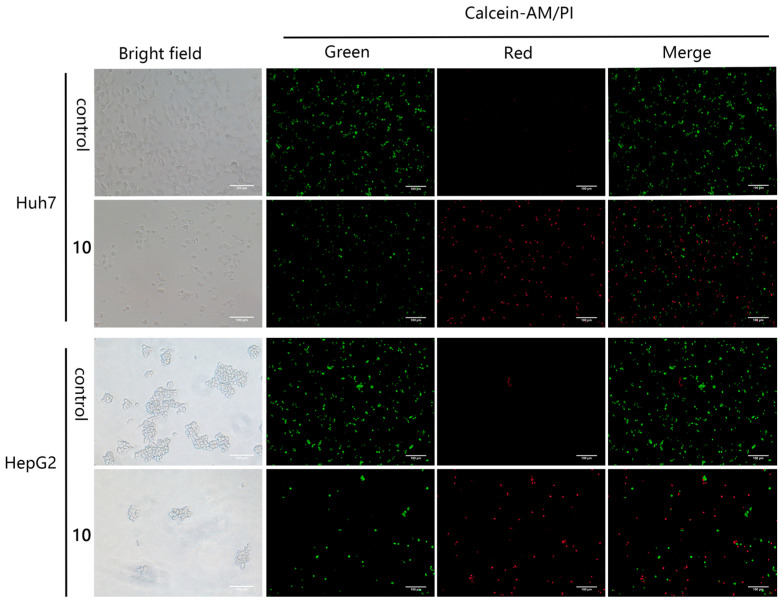
Live/dead cell staining of Huh7 and HepG2 cells treated with compound **10**. Scale bar: 100 μm.

The effects of different concentrations of compound **10** on two types of human HCC cell lines (Huh7 and HepG2) were detected by CCK-8 assay. The results indicated that **10** could dose-dependently decrease the viability of the two HCC cell lines (Figure 2A,B). In addition, **10** could also inhibit the viability of Huh7 cells in time- and dose-dependent manners (Figure 2C,D). The live/dead cell staining assay (Calcein-AM/PI double staining kit) was also employed to evaluate the in vitro cytotoxicity of **10**. In the control, a bright green fluorescence (calcein AM for live cells probe) and a few red dots with fluorescence (PI for dead cells probe) were found in Huh7 and HepG2 cells (Figure 3). A decreased number of live cells for both Huh7 and HepG2 was obviously found in 10, whereas that of dead cells increased, compared with the control. Thus, the fluorescence imaging results were in agreement with those of the cytotoxicity assay.

**Figure 4:**
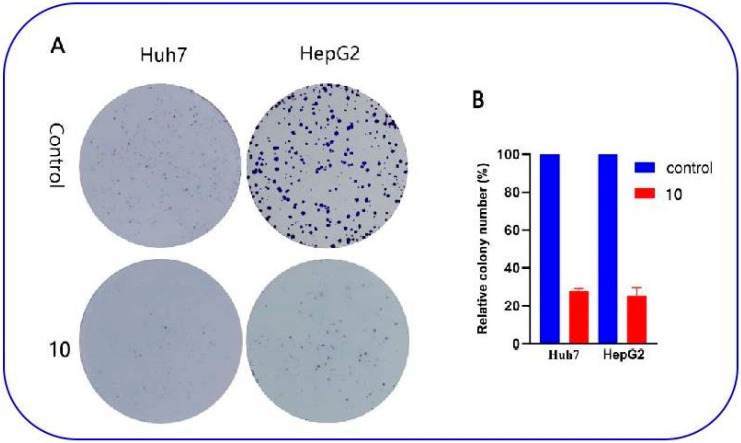
Compound **10** suppressed the colony formations of Huh7 and HepG2 cells. (**A**,**B**) Huh7 and HepG2 cells were treated with **10** for 14 d, and dyed with 0.1% crystal violet. The relative colony numbers are represented as a histogram.

The colony-forming assay is one of the effective methods to measure cell proliferation. When a single cell lasts for more than six generations in vitro, the descendants of the cell population are called clones. At this time, each clone contains more than 50 cells, and the size is between 0.3 and 1.0 mm. By counting the clone formation rate, the proliferation potential of a single cell can be quantitatively analyzed, and the proliferation ability and independent survival ability of the cell can be understood. Consequently, this work performed a clone-forming assay to investigate whether compound **10** inhibited proliferation. Huh7 and HepG2 cells were first incubated using **10** for a 24 h period, respectively, and followed by 14 d culture to observe a visible colony. According to Figure 4, Huh7 and HepG2 cells had significantly suppressed clone-forming capacity after **10** exposure, which was consistent with the CCK-8 assays.

#### 2.2.3. Compound **10** Promoted Cell Apoptosis of Huh7 and HepG2 Cells

**Figure 5:**
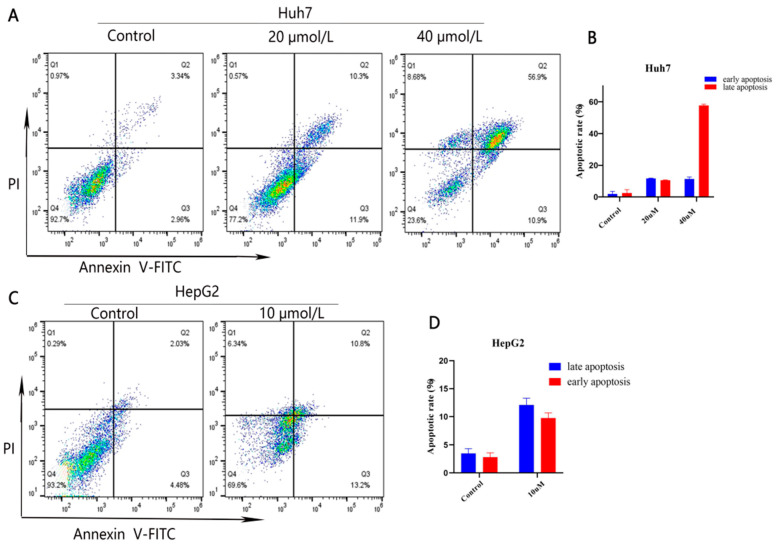
Compound **10** induced apoptosis of Huh7 and HepG2 cells. (**A**,**C**) Huh7 and HepG2 cells were treated with **10** for 24 h. The apoptosis was analyzed by flow cytometry. (**B**,**D**) The apoptotic rates are represented as a histogram.

In order to investigate the proapoptotic effect of compound **10**, Huh7 and HepG2 cells were stained with Annexin V-FITC and PI probe, and subjected to flow cytometry analysis. Both cells were incubated with different concentrations of **10** for 24 h. In comparison to the control group, all treatment groups of **10** significantly induced apoptosis of Huh7 and HepG2 cells (Figure 5). The percentages of total apoptotic cells increased significantly from 6.3% (control, Huh7 cells) and 6.51% (control, HepG2 cells) to 67.8% (40 μM, Huh7 cells) and 24% (10 μM, HepG2 cells), respectively. Compound **10** induced approximately 30% late apoptosis of Huh7 cells at concentrations of 40 μM, and late apoptosis was more obvious. The results established that compound **10** could significantly induce Huh7 and HepG2 cell apoptosis.

#### 2.2.4. Compound **10** Arrested the Cell Cycle in the S Phase in Huh7 and HepG2 Cells

**Figure 6:**
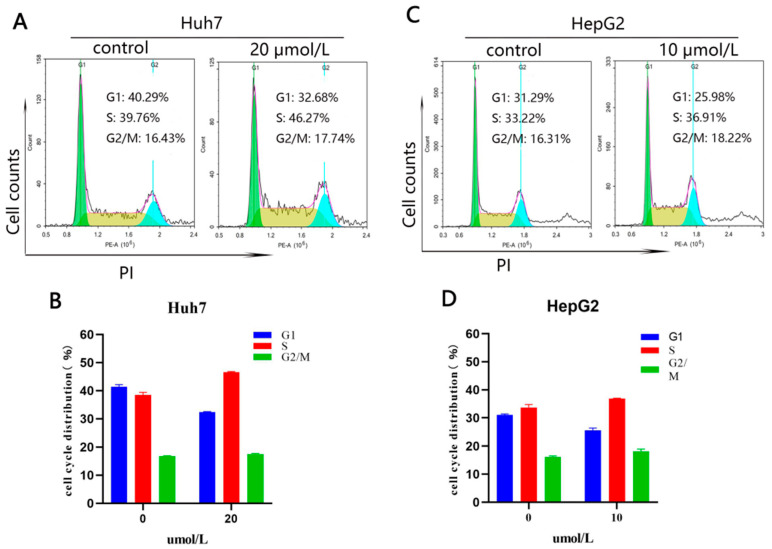
Compound **10** induced S phase arrest in Huh7 and HepG2 cells. (**A**,**C**) Huh7 and HepG2 cells were incubated with **10** for 24 h, respectively. The distribution of the cell cycle was analyzed by flow cytometry. (**B**,**D**) The percentage of cell cycle distribution is represented as a histogram.

The cell cycle plays an important role in the operation and development of cell life due to its regulation of the division and duplication of DNA. Therefore, blocking the cell cycle is considered an effective way to eliminate tumor cells. To demonstrate the antiproliferative effect of compound **10** on the cell cycle process, flow cytometry was used to monitor PI staining treatment with **10** for 24 h; this led to an increased percentage of cells in the S phase from 39.76% and 33.22% of the untreated control to 46.27% and 36.91%, respectively (Figure 6A,B and Figure 6C,D). This result demonstrated that **10** arrested Huh7 and HepG2 cell cycle progression in the S phase. 

#### 2.2.5. Effect of Compound **10** on Cell Migration

**Figure 7:**
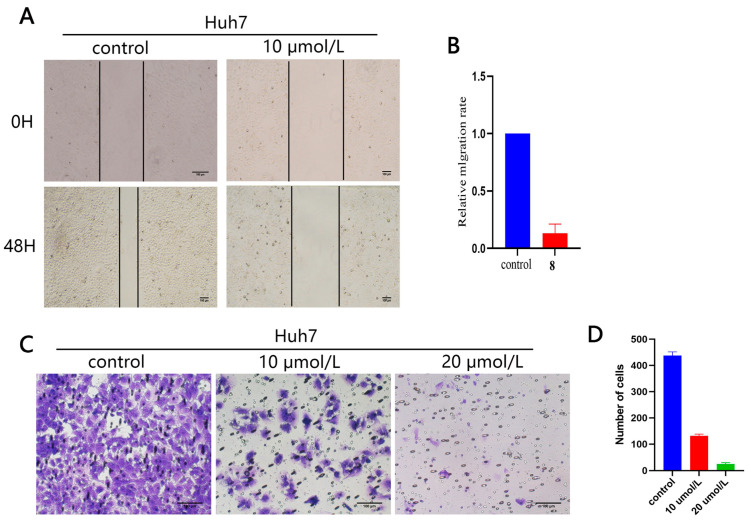
Experimental results of the migration of cells incubated in compound **10**. (**A**) Wound healing assay results of Huh7 cells incubated in compounds for 0 h and 48 h (scale bar = 100 μm, magnification 4×). (**B**) Statistical analysis of the scratch experiment and the healing rate (%) = (scratch area at 0 h -scratch area at 24 h)/scratch area at 0 h ×100%. (**C**) Transwell migration result of Huh7 cells treated with different concentrations of compound **10** for 48 h (scale bar = 100 μm, magnification 20×). (**D**) Statistical results of Transwell migration tests (*n* = 3).

Migration is one of the essential links in the process of tumor cell metastasis. For tumor cells to separate from the mother tumor, cross the blood vessel wall, and invade the surrounding normal tissue, they need some exercise ability. Tumor cells that are highly metastatic usually have high motility. The cell scratch method and transwell migration are simple methods to determine the migration, movement, and repair ability of cells. Thus, the effects of **10** on cell migration were further investigated. As shown in Figure 7A,B, **10** significantly inhibited the rate of wound healing in Huh7 cells after treatment at the indicated concentrations for 48 h. The number of cells that went through the membrane in transwell assays also decreased in a dose-dependent manner (Figure 7C,D). The above results suggested that **10** could effectively suppress the migration of Huh7 cells in a dose-dependent manner.

#### 2.2.6. ADME Prediction

Many drug molecule candidates remain in phase studies without a drug molecule due to poor ADME properties. Performing theoretical ADME calculations for designed and newly synthesized compounds may aid the progression of these compounds in advanced in vitro and in vivo studies [33,34]. Therefore, some properties of the designed compounds, such as physicochemical, lipophilicity, water-solubility, pharmacokinetics, drug-likeness, and medicinal chemistry properties, were calculated using SwissADME online tools, and some of the most active properties of **10** are given in Table 2. The drug-likeness assessment of **10** was performed by predicting Lipinski’s rule of five, which includes molecular weights (MW < 500), lipophilicity (log Po/w < 5), number of hydrogen bond acceptors (HBA ≤ 10), and number of hydrogen bond donors (HBD ≤ 5) to determine the “drug-likeness” of **10**. Its solubility in water is fine and favorable. Its gastrointestinal absorption was calculated as high and not able to pass through the blood–brain barrier. The findings of ADME showed that **10** has very good drug-like properties.

## 3. Materials and Methods

### 3.1. General Experimental Procedures

The 1D and 2D NMR experiments were performed on a Bruker AV-400 (Bruker Corporation, Zurich, Switzerland) instrument with tetramethylsilane as the internal standard. HR-ESI-MS spectra were acquired on a Bruker Daltonics Apex-Ultra 7.0 T (Bruker Corporation, Billerica, MA, USA) and a Q-TOF Ultima Global GAA076 LC mass spectrometer. Single-crystal data were measured with an Agilent Gemini Ultra X-ray single-crystal diffractometer (Cu Kα radiation). Preparative HPLC was conducted using an Agilent 1260 prep-HPLC system with a Waters C18 analytical HPLC column (4.6 × 250 mm, 5 μm) and a semipreparative column (9.4 × 250 mm, 7 μm). Sephadex LH-20 (Pharmacia Co. Ltd., Sandwich, UK) and silica gel (200–300 and 300–400 mesh, Qingdao Marine Chemical Inc., Qingdao, China) were used for column chromatography (CC). All solvents were purchased from Xilong Chemical Reagent Factory (Guangzhou, China). The flowers of *Sphagneticola trilobata* were collected from Haikou County, Hainan Province, China, in August 2018, and identified by Professor Qiong-Xin Zhong, School of Life Science, Hainan Normal University.

### 3.2. The Separation of SLs ***1*** and ***2***

The isolation process of **1** and **2** is shown in the supporting information.

### 3.3. General Procedure for the Synthesis of ***1*** Hydrolysis Derivatives ***3***–***4***

In a 25 mL round-bottomed flask, 712 mg (1.157 mmol) of **1**, 2 mL of THF, and 2 mL of dilute HCl (2 mol/L) were added sequentially. The reaction was heated under reflux at 45 °C for 26 h. After the reaction was complete (CHCl_3_:acetone = 6:1), the reaction was detected by TLC. The pH was adjusted to 6–7 with potassium carbonate solution, and products **3** and **4** were separated by CC (CHCl_3_:acetone = 8:1).

(3aS,4R,4aS,8S,8aS,9R,9aS)-4-(isobutyryloxy)-8a-methyl-3,5-dimethylene-2-oxododecahydronaphtho[2,3-b]furan-8,9-diyl diacetate (compound **3**). Yield: 4 mg (6%), purity 93%. C_23_H_30_O_8_, white amorphous powder. ^1^H NMR (400 MHz, CDCl_3_) δ: 6.18 (1H, d, J = 3.6 Hz, H-13), 5.49 (1H, d, J = 3.2 Hz, H-13), 5.42 (1H, dd, J = 8.0, 11.6 Hz, H-6), 5.30 (1H, d, J = 2.4 Hz, H-9), 5.23 (1H, dd, J = 4.4, 12.0 Hz, H-1), 4.96 (1H, t, J = 2.0 Hz, H-14), 4.85 (1H, dd, J = 2.4, 9.6 Hz, H-8), 4.76 (1H, t, J = 2.0 Hz, H-14), 3.43 (1H, m, H-7), 2.55 (1H, m, H-2″), 2.38 and 2.20 (each 1H, m, H-3), 2.16 (1H, d, J = 11.9 Hz, H-5), 1.95 (3H, s, H-2‴), 1.93 (3H, s, H-2′), 1.87 and 1.44 (each 1H, m, H-2), 1.21 (3H, s, H-15), 1.16 (6H, d, J = 7.0 Hz, H-3″, 4″); ^13^C NMR (100 MHz, CDCl_3_) δ: 175.8 (C-1″), 170.5 (C-1′), 169.3 (C-12), 169.1 (C-1‴), 141.1 (C-4), 136.8 (C-11), 120.2 (C-13), 117.4 (C-14), 73.4 (C-8), 73.0 (C-9), 70.6 (C-6), 70.4 (C-1), 55.5 (C-5), 44.6 (C-7), 40.7 (C-10), 34.1 (C-2″), 30.1 (C-3), 29.7 (C-15), 26.6 (C-2), 21.1 (C-2′), 20.5 (C-2‴), 19.5 (C-3″), 19.1 (C-4″). HRESIMS *m*/*z* 457.1822 [M+Na]^+^ (calcd. for C_23_H_30_O_8_Na, 457.1823).

(3aS,4R,4aS,8S,8aS,9R,9aS)-4-hydroxy-5,8a-dimethyl-3-methylene-2-oxo-2,3,3a,4,4a,7,8,8a,9,9a-decahydronaphtho[2,3-b]furan-8,9-diyl diacetate (compound **4**). Yield: 9 mg (16%), purity 97%. C_19_H_24_O_7_, white amorphous powder. ^1^H NMR (400 MHz, CDCl_3_) δ: 6.20 (1H, d, J = 2.4 Hz, H-13), 6.00 (1H, d, J = 2.4 Hz, H-13), 5.51 (1H, m, H-3), 5.28 (1H, d, J = 2.8 Hz, H-9), 5.08 (1H, dd, J = 6.0, 9.6 Hz, H-1), 4.85 (1H, dd, J = 2.8, 9.6 Hz, H-8), 4.19 (1H, m, H-6), 3.25 (1H, m, H-7), 2.48, 1.97 (2H, m, H-2), 2.11 (1H, d, J = 4.4 Hz, OH), 1.95 (3H, s, H-2′), 1.91 (3H, s, H-14), 1.86 (3H, s, H-2″), 1.83 (1H, s, H-5), 1.13 (3H, s, H-15); ^13^C NMR (100 MHz, CDCl_3_) δ: 171.1 (C-1′), 170.1 (C-12), 169.1 (C-1″), 137.5 (C-11), 131.1 (C-4), 122.4 (C-3), 121.1 (C-13), 75.1 (C-6), 73.9 (C-8), 73.7 (C-9), 68.1 (C-1), 54.1 (C-5), 47.2 (C-7), 39.0 (C-10), 28.0 (C-2), 25.7 (C-14), 21.3 (C-2′), 20.6 (C-2″), 18.8 (C-15). HRESIMS *m*/*z* 387.1407 [M+Na]^+^ (calcd. for C_19_H_24_O_7_+Na, 387.1414).

### 3.4. General Procedure for the Synthesis of ***1*** Hydrolysis Derivative ***5***

Add 125.0 mg (0.277 mmol) 1 to a 50 mL round bottom flask and dissolve in 6 mL THF and 10 mL H_2_O. Add 4 mL KOH solution (0.5 mol/L) slowly at 0 °C, stirring for 2 h. After the reaction was completely detected by TLC (Petroleum ether:acetone = 1.5:1), the mixture was adjusted to pH 2–3 with 10% dilute HCl, and product **5** was purified by CC (petroleum ether:acetone = 3:1).

(3aS,4S,4aS,5S,8S,8aS,9R,9aS)-5,8,9-trihydroxy-5,8a-dimethyl-3-methylene-2-oxododecahydronaphtho[2,3-b]furan-4-yl isobutyrate (compound **5**). Yield: 73.0mg (71%), purity 92%. C_19_H_28_O_7_, white amorphous powder. ^1^H NMR (400 MHz, CDCl_3_) δ: 6.25 (1H, d, J = 3.2 Hz, H-13), 5.68 (1H, d, J = 2.8 Hz, H-13), 5.65 (1H, t, J = 4.8 Hz, H-6), 4.90 (1H, dd, J = 4.0, 10.0 Hz, H-8), 4.03 (1H, dd, J = 2.8, 6.8 Hz, H-1), 3.96 (1H, d, J = 3.6 Hz, H-9), 3.27 (1H, m, H-7), 2.52 (1H, m, H-2′), 1.97 and 1.62 (each 1H, m, H-2), 1.80 (1H, d, J = 4.4 Hz, H-5), 1.77 and 1.52 (each 1H, m, H-3), 1.17 (3H, s, H-15), 1.16 (3H, s, H-14), 1.15 (6H, d, J = 7.0 Hz, H-3′, 4′); ^13^C NMR (100 MHz, CDCl_3_) δ: 176.4 (C-1′), 171.0 (C-12), 135.4 (C-11), 123.2 (C-13), 75.7 (C-8), 71.9 (C-6), 70.0 (C-10), 69.7 (2C, C-1, 9), 51.5 (C-5), 42.6 (C-7), 42.5 (C-10), 34.5 (C-3), 34.4 (C-2′), 29.4 (C-2), 25.7 (C-14), 20.7 (C-15), 18.9 (C-3′), 18.8 (C-4′). HRESIMS *m*/*z* 391.1723 [M+Na]^+^ (calcd. for C_19_H_28_O_7_+Na, 391.1727).

### 3.5. General Procedure for the Synthesis of ***1*** Hydrolysis Derivatives ***6***–***9***

An amount of 297 mg (0.657 mmol) of compound **1** and 88 mg of NaBH4 were dissolved in 50 mL of CH_3_CN and stirred at 60 °C for 26.5 h. After the reaction was complete (CHCl_3_: acetone = 6: 1) as detected by TLC, the pH of the mixture was adjusted to 6–7 with 10% dilute HCl, extracted with EtOAc for three times, dried with anhydrous sodium sulfate, concentrated under reduced pressure, and prepared by HPLC to obtain compounds **6** and **7**. Subsequently, 23.3 mg (0.051 mmol) of **6** and 22.6 mg KOH were dissolved in 5 mL of MeOH, stirred at room temperature for 6 h, and the reaction was detected by TLC (CHCl_3_:acetone = 3:1). The products **8** and **9** were purified by CC (CHCl_3_:acetone = 6:1).

*(3R,3aS,4S,4aS,5S,8S,8aS,9R,9aS)-5-hydroxy-4-(isobutyryloxy)-3,5,8a-trimethyl-2-oxododecahydronaphtho[2,3-b]furan-8,9-diyl diacetate (compound **6**).* Yield: 86 mg (29%), purity 98%. C_23_H_34_O_9_, white amorphous powder. ^1^H-NMR (400 MHz, Methanol-d_4_) δ: 5.83 (1H, dd, J = 9.2, 12.0 Hz, H-6), 5.52 (1H, dd, J = 8.8, 8.8 Hz, H-1), 5.16 (1H, d, J = 2.4 Hz, H-9), 4.99 (1H, dd, J = 2.4, 10.0 Hz, H-8), 2.79 (1H, m, H-11), 2.57 (1H, m, 2″), 2.51 (1H, m, H-7), 2.23, 1.42 (2H, m, H-2), 1.98 (3H, s, H-2′),1.92 (3H, s, H-2‴), 1.96, 1.67 (2H, m, H-3), 175 (1H, d, J = 11.6 Hz, H-5), 1.38 (3H, s, H-15), 1.31 (3H, s, H-14), 1.20 (6H, d, J = 6.8 Hz, H-3″, 4″), 1.16 (3H, d, J = 7.6 Hz, H-13); ^13^C-NMR (100 MHz, Methanol-d_4_) δ: 181.5 (C-12), 178.1 (C-1″), 172.4 (C-1′), 170.9 (C-1‴), 75.9 (C-8), 75.2 (C-9), 74.3 (C-6), 70.7 (C-4), 70.2 (C-1), 54 (C-5), 49.3 (C-7), 41.5 (C-10), 41.2 (C-11), 36 (C-3), 35.6 (C-2″), 32.9 (C-15), 23.2 (C-2), 22 (C-14), 21.3 (C-2′), 21 (C-2‴), 19.7 (C-3″), 18.8 (C-4″), 17.9 (C-13). HRESIMS *m*/*z* 477.2097 [M+Na]^+^ (calcd. for C_23_H_34_O_9_+Na, 477.2095).

*(3R,3aS,4S,4aS,5S,8S,8aR,9R,9aS)-8-acetoxy-5,9-dihydroxy-3,5,8a-trimethyl-2-oxododecahydronaphtho[2,3-b]furan-4-yl isobutyrate (compound **7**).* Yield: 80 mg (30%), purity 97%. C_21_H_32_O_8_, white amorphous powder. ^1^H NMR (400 MHz, DMSO-d_6_) δ: 5.61 (1H, dd, J = 8.8, 6.8 Hz, H-6), 5.26 (1H, dd, J = 7.2, 14.4 Hz, H-1), 4.79 (1H, dd, J = 2.8, 10.0 Hz, H-8), 3.53 (1H, d, J = 2.8 Hz, H-9), 2.75 (1H, m, H-11), 2.47 (1H, m, H-2″), 2.29 (1H, m, H-7), 2.18, 1.31 (2H, m, H-2), 1.95 (3H, s, H-2′), 1.79, 1.51 (2H, m, H-3), 1.55 (1H, d, J = 8.8 Hz, H-5), 1.24 (3H, s, H-15), 1.11 (6H, d, J = 7.2 Hz, H-3″, 4″), 1.08 (3H, s, H-14), 1.07 (3H, s, H-13); ^13^C NMR (100 MHz, DMSO-d_6_) δ: 179.6 (C-12), 174.9 (C-1″), 169.6 (C-1′), 75 (C-8), 71.7 (C-6), 71.6 (C-9), 70.8 (C-1), 68.6 (C-4), 51.9 (C-5), 47.3 (C-7), 40.1 (C-10), 39.1 (C-11), 34.5 (C-3), 33.5 (C-2″), 31.5 (C-15), 22.7 (C-2), 21.3 (C-14), 21.1 (C-2′), 19 (C-3″), 18.3 (C-4″), 16.5 (C-13). HRESIMS *m*/*z* 435.1987 [M+Na]^+^ (calcd. for C_21_H_32_O_8_+Na, 435.1989).

(3R,3aS,4S,4aS,5S,8S,8aS,9R,9aS)-4,5-dihydroxy-3,5,8a-trimethyl-2-oxododecahydronaphtho[2,3-b]furan-8,9-diyl diacetate (compound **8**). Yield: 3.5 mg (18%), purity 96%. C_19_H_28_O_8_, white amorphous powder. ^1^H-NMR (400 MHz, Methanol-d_4_) δ: 5.48 (1H, dd, J = 8.4, 9.2 Hz, H-1), 5.13(1H, d, J = 2.8 Hz, H-9), 4.94 (1H, dd, J = 2.8, 10.4 Hz, H-8), 4.34 (1H, dd, J = 9.6, 11.6 Hz, H-6), 2.61 (1H, m, H-11), 2.37 (1H, m, H-7), 2.22, 1.33 (2H, m, H-2), 1.93 (3H, s, H-2′), 1.90 (3H, s, H-2″), 1.71,1.30 (2H, m, H-3), 1.45 (3H, s, H-14), 1.44 (1H, d, J = 11.2 Hz, H-5), 1.36 (3H, d, J = 7.6 Hz, H-13), 1.27 (3H, s, H-15); ^13^C NMR (100 MHz, Methanol-d_4_) δ: 182.2 (C-12), 172.5 (C-1″), 171 (C-1′), 76.2 (C-8), 75.4 (C-9), 73.5 (C-1), 72.1 (C-6), 70.4 (C-4), 56.4 (C-5), 50.4 (C-7), 42.8 (C-10), 41.5 (C-11), 35.6 (C-2), 33.5 (C-15), 23.3 (C-2), 22.2 (C-14), 21.3 (C-2′), 21 (C-2″), 18.2 (C-13). HRESIMS *m*/*z* 407.1669 [M+Na]^+^ (calcd. for C_19_H_28_O_8_+Na, 407.1676).

(3R,3aS,4S,4aS,5S,8S,8aS,9R,9aS)-5,8,9-trihydroxy-3,5,8a-trimethyl-2-oxododecahydronaphtho[2,3-b]furan-4-yl isobutyrate (compound **9**). Yield: 6.0 mg (31%), purity 98%. C_19_H_30_O_7_, white amorphous powder. ^1^H NMR (400 MHz, Methanol-d_4_) δ: 5.62 (1H, dd, J = 2.8, 3.2 Hz, H-6), 4.89 (1H, dd, J = 2.8, 10.0 Hz, H-8), 3.84 (1H, dd, J = 4.0, 5.2 Hz, H-1), 3.70 (1H, d, J = 3.6 Hz, H-9), 3.03 (1H, m, H-11), 2.54 (1H, m, H-2′), 2.38 (1H, m, H-7), 2.27, 1.63 (2H, m, H-2), 1.92, 1.50 (2H, m, H-3), 1.79 (1H, d, J = 3.2 Hz, H-5), 1.28 (3H, s, H-14), 1.24 (3H, s, H-13), 1.16 (3H, s, H-15), 1.15 (6H, d, J = 6.8 Hz, H-3′, 4′); ^13^C NMR (100 MHz, Methanol-d_4_) δ: 182.1 (C-12), 177.1 (C-1′), 76.7 (C-8), 72.9 (C-9), 72.6 (C-4), 71.9 (C-6), 70.3 (C-1), 52.0 (C-5), 48.6 (C-7), 43.3 (C-10), 39.2 (C-11), 35.4 (C-2′), 35.1 (C-3), 30.0 (C-15), 27.8 (C-2), 22.3 (C-14), 19.2 (C-3′), 19.1 (C-4′), 16.1 (C-13). HRESIMS *m*/*z* 393.1875 [M+Na]^+^ (calcd. for C_19_H_30_O_7_+Na, 393.1883).

### 3.6. General Procedure for the Synthesis of ***2*** Hydrolysis Derivatives ***10***–***12***

Referring to the synthesis method of **3** and **4**, compounds **10** and **11** were obtained by CC purification (CHCl_3_:acetone = 3:1). For further optimization of acid conditions, we replaced the previous acid with a dilute H_2_SO_4_ solution (8 mol/L) and refluxed the mixture at 45 °C for 10 h. After the reaction was completed (CHCl_3_:acetone = 6:1), the products **10**, **11**, and **12** were separated by CC (CHCl_3_:acetone = 8:1).

(3aS,4R,4aR,8S,8aS,9R,9aS)-9-acetoxy-8-hydroxy-5,8a-dimethyl-3-methylene-2-oxo-2,3,3a,4,4a,7,8,8a,9,9a-decahydronaphtho[2,3-b]furan-4-yl isobutyrate (compound **10**). Yield: 11.2 mg (6%), purity 93%. C_21_H_28_O_7_, white amorphous powder. ^1^H NMR (400 MHz, CDCl_3_) δ: 6.32 (1H, d, J = 4 Hz, H-13), 5.77 (1H, d, J = 3.2 Hz, H-13), 5.53 (1H, dd, J = 1.6, 4.0 Hz, H-6), 5.41 (1H, br s, H-3), 5.31 (1H, d, J = 4.4 Hz, H-9), 5.03 (1H, dd, J = 4.4, 8.0 Hz, H-8), 3.43 (1H, dd, J = 6.0, 10.4 Hz, H-1), 3.31 (1H, m, H-7), 2.56 (1H, m, H-2′), 2.48 (1H, H-5), 2.24, 2.07 (2H, m, H-2), 2.03 (3H, s, H-2″), 1.60 (3H, d, J = 1.2 Hz, H-14), 1.17 (6H, d, J = 7.2 Hz, H-3′, 4′), 1.11 (3H, s, H-15); ^13^C NMR (100 MHz, CDCl_3_) δ: 176.6 (C-1′), 172.3 (C-1″), 169.6 (C-12), 134.9 (C-11), 130.2 (C-4), 122.4 (C-3), 119.8 (C-13), 72.8 (C-8), 72.2 (C-9), 69.7 (C-6), 68.4 (C-1), 43.5 (C-7), 41.4 (C-10), 37.9 (C-5), 34.4 (C-2′), 29.7 (C-2), 20.7 (C-2″), 20.4 (C-14), 19.0 (C-3′), 18.8 (C-4′), 11.0 (C-15). HRESIMS *m*/*z* 415.1725 [M+Na]^+^ (calcd. for C_21_H_28_O_7_+Na, 415.1727).

(3aS,4S,4aR,5S,8S,8aS,9R,9aS)-9-acetoxy-5,8-dihydroxy-5,8a-dimethyl-3-methylene-2-oxododecahydronaphtho[2,3-b]furan-4-yl isobutyrate (compound **11**). Yield: 82.2 mg (48%), purity 95%. C_21_H_30_O_8_, white amorphous powder. ^1^H NMR (400 MHz, Methanol-d_4_) δ: 6.18 (1H, d, J = 4.0 Hz, H-13), 5.90 (1H, dd, J = 1.6, 3.2 Hz, H-6), 5.72 (1H, d, J = 4.0 Hz, H-13), 5.34 (1H, d, J = 4.4 Hz, H-9), 5.08 (1H, dd, J = 4.4, 8.4 Hz, H-8), 3.39 (1H, dd, J = 6.4, 8.4 Hz, H-1), 3.31 (1H, m, H-7), 2.61 (1H, m, H-2′), 1.94 (3H, s, H-2″), 1.86 (1H, d, J = 3.2 Hz, H-5), 1.68, 1.50 (2H, m, H-3), 1.64 (2H, m, H-2), 1.34 (3H, s, H-14), 1.30 (3H, s, H-15), 1.21 (6H, d, J = 7.0 Hz, H-3′, 4′); ^13^C NMR (100 MHz, Methanol-d_4_) δ: 177.5 (C-1′), 171.8 (C-12), 170.9 (C-1″), 136.8 (C-11), 119.0 (C-13), 74.5 (C-8), 73.6 (C-9), 72.2 (C-4), 72.1 (C-1), 70.0 (C-6), 45.8 (C-7), 44.5 (C-5), 43.7 (C-3), 43.1 (C-10), 35.7 (C-2′), 28.6 (C-14), 26.3 (C-2), 20.5 (C-2″), 19.3 (C-3′), 18.8 (C-4′), 13.8 (C-15). HRESIMS *m*/*z* 428.2272 [M+NH_4_]^+^ (calcd. for C_21_H_30_O_8_+NH_4_, 428.2278).

(3aS,4S,4aR,5S,8S,8aS,9R,9aS)-5,8,9-trihydroxy-5,8a-dimethyl-3-methylene-2-oxododecahydronaphtho[2,3-b]furan-4-yl isobutyrate (compound **12**). Yield: 63 mg (43%), purity 94%. C_19_H_28_O_7_, white amorphous powder. ^1^H NMR (400 MHz, Methanol-d_4_) δ: 6.56 (1H, d, J = 1.2 Hz, H-13), 5.82 (1H, d, J = 1.2 Hz, H-13), 5.41 (1H, dd, J = 2.4, 3.6 Hz, H-6), 4.51 (1H, d, J = 2.4 Hz, H-9), 4.25 (1H, dd, J = 2.4, 4 Hz, H-8), 3.79 (1H, dd, J = 6.4, 9.6 Hz, H-1), 3.16 (1H, m, H-7), 2.62 (1H, m, H-2′), 1.72, 1.60 (2H, m, H-2), 1.67, 1.44 (2H, m, H-2), 1.35 (1H, d, J = 2.8 Hz, H-5), 1.29 (3H, s, H-14), 1.26 (3H, s, H-15), 1.22 (6H, d, J = 7.2 Hz, H-3′, 4′); ^13^C NMR (100 MHz, Methanol-d_4_) δ: 177.3 (C-1′), 166.5 (C-12), 135.2 (C-11), 132.8 (C-13), 86.1 (C-9), 76.1 (C-6), 71.8 (C-1), 71.6 (C-4), 62.2 (C-8), 47.2 (C-7), 45.3 (C-5), 44.6 (C-10), 42.8 (C-3), 35.7 (C-2′), 28.8 (C-2), 25 (C-14), 19.5 (C-3′), 19 (C-4′), 15.2 (C-15). HRESIMS *m*/*z* 386.2166 [M+NH_4_]^+^ (calcd. for C_19_H_28_O_7_+NH_4_, 386.2173).

### 3.7. General Procedure for the Synthesis of ***2*** Hydrolysis Derivatives ***13***–***16***

According to the synthesis of **6** and **7**, compounds **13** and **14** were prepared by HPLC. According to the synthesis of **8** and **9**, compounds **15** and **16** were purified by CC (CHCl_3_:acetone = 3:1).

(3R,3aS,4S,4aR,5S,8S,8aS,9R,9aS)-5-hydroxy-4-(isobutyryloxy)-3,5,8a-trimethyl-2-oxododecahydronaphtho[2,3-b]furan-8,9-diyl diacetate (compound **13**). Yield: 131.6 mg (43%), purity 95%. C_23_H_34_O_9_, white amorphous powder. ^1^H NMR (400 MHz, DMSO-d_6_) δ: 5.50 (1H, dd, J = 1.2, 2.8 Hz, H-6), 5.05 (1H, d, J = 4.4 Hz, H-9), 4.94 (1H, dd, J = 4.4, 7.6 Hz, H-8), 4.55 (1H, dd, J = 6.4, 9.2 Hz, H-1), 2.56, 1.58 (2H, m, H-2), 2.53 (1H, m, H-2″), 2.54 (1H, m, H-11), 2.34 (1H, m, H-7), 1.93 (1H, d, J = 2.8 Hz, H-5), 1.92 (3H, s, H-2′), 1.90 (3H, s, H-2‴), 1.61, 1.50 (2H, m, H-3), 1.31 (3H, s, H-15), 1.19 (3H, s, H-14), 1.16 (3H, d, J = 6.8 Hz, H-13), 1.11 (6H, d, J = 7.2 Hz, H-3″, 4″); ^13^C NMR (100 MHz, DMSO-d_6_) δ: 178.4 (C-12), 174.9 (C-1″), 169.8 (C-1′), 169.7 (C-1‴), 73.0 (C-1), 71.5 (C-8), 70.8 (C-9), 69.6 (C-4), 67.6 (C-6), 47.2 (C-7), 42.3 (C-5), 41.6 (C-3), 41.0 (C-10), 35.7 (C-2″), 33.7 (C-11), 25.5 (C-14), 23.9 (C-2), 20.8 (C-2′), 20.2 (C-2‴), 18.6 (C-3″), 18.2 (C-4″), 14.2 (C-13), 13.9 (C-15). HRESIMS *m*/*z* 477.2099 [M+Na]^+^ (calcd. for C_23_H_34_O_9_+Na, 477.2095).

(3R,3aS,4S,4aR,5S,8S,8aS,9R,9aS)-5,8,9-trihydroxy-3,5,8a-trimethyl-2-oxododecahydronaphtho[2,3-b]furan-4-yl isobutyrate (compound **14**). Yield: 3 mg (1%), purity 98%. C_19_H_30_O_7_, white amorphous powder. ^1^H NMR (400 MHz, Methanol-d_4_) δ: 5.57 (1H, dd, J = 1.2, 2.8 Hz, H-6), 4.69 (1H, dd, J = 4.0, 7.6 Hz, H-8), 4.02 (1H, dd, J = 4.8, 9.6 Hz, H-1), 3.85 (1H, d, J = 3.6 Hz, H-9), 2.56 (1H, m, H-2′), 2.47 (1H, m, H-7), 2.28 (1H, m, H-11), 2.02 (1H, d, J = 2.8 Hz, H-5), 1.70, 1.54 (2H, m, H-3), 1.68, 1.64 (2H, m, H-2), 1.31 (3H, s, H-15), 1.23 (3H, d, J = 6.4 Hz, H-13), 1.20 (3H, s, H-14), 1.20 (6H, d, J = 6.8 Hz, H-3′, 4′); ^13^C NMR (100 MHz, Methanol-d_4_) δ: 182.7 (C-12), 177.5 (C-1′), 76.2 (C-8), 72.3 (C-1), 71.8 (C-9), 71.7 (C-4), 70.4 (C-6), 50.2 (C-5), 44.5 (C-10), 43.5 (C-3), 42.7 (C-7), 38.8 (C-11), 35.7 (C-2′), 28.6 (C-2), 26.2 (C-14), 18.4 (C-3′), 18.8 (C-4′), 15 (C-13), 13.9 (C-15). HRESIMS *m*/*z* 369.1920 [M-H]^-^ (calcd. for C_19_H_29_O_7_, 369.1907).

(3R,3aS,4S,4aR,5S,8S,8aS,9R,9aS)-4,5-dihydroxy-3,5,8a-trimethyl-2-oxododecahydronaphtho[2,3-b]furan-8,9-diyl diacetate (compound **15**). Yield: 17.2 mg (41%), purity 95%. C_19_H_28_O_8_, white amorphous powder. ^1^H NMR (400 MHz, DMSO-d_6_) δ: 5.19 (1H, d, J = 4.4 Hz, H-9), 4.97 (1H, dd, J = 4.4, 8.8 Hz, H-8), 4.67 (1H, dd, J = 5.6, 10.0 Hz, H-1), 4.57 (1H, dd, J = 1.6, 2.8 Hz, H-6), 2.54 (1H, m, H-11), 2.48 (1H, m, H-7), 1.98 (3H, s, H-2′), 1.97 (3H, s, H-2″), 1.79, 1.60 (1H, m, H-3), 1.75, 1.74 (1H, m, H-2), 1.69 (1H, d, J = 2.8 Hz, H-5), 1.58 (3H, s, H-15), 1.44 (3H, s, H-14), 1.25 (3H, d, J = 6.4 Hz, H-13); ^13^C NMR (100MHz, DMSO-d_6_) δ: 179.2 (C-12), 169.9 (C-1″), 168.7 (C-1′), 73.4 (C-1), 72.1 (C-8), 71.6 (C-9), 70.4 (C-4), 64.1 (C-6), 51.9 (C-7), 48.6 (C-5), 43.1 (C-10), 41.7 (C-3), 40.9(C-11), 35.8(C-14), 25.6 (C-2), 20.8 (C-2″), 20.3 (C-2′), 14.9 (C-15), 14.3 (C-13). HRESIMS *m*/*z* 402.2115 [M+NH_4_]^+^ (calcd. for C_19_H_28_O_8_+NH_4_, 402.2122).

*(3R,3aS,4S,4aR,5S,8S,8aS,9R,9aS)-4,5,8,9-tetrahydroxy-3,5,8a-trimethyldecahydronaphtho[2,3-b]furan-2(3H)-one (compound **16**).* Yield: 3.6 mg (11%), purity 94%. C_15_H_24_O_6_, white amorphous powder. ^1^H NMR (400 MHz, Methanol-d_4_) *δ*: 4.75 (1H, dd, *J* = 8.0, 4.0 Hz, H-8), 4.46 (1H, dd, *J* = 2.8, 1.6 Hz, H-6), 3.98 (1H, dd, *J* = 9.6, 7.2 Hz, H-1), 3.81 (1H, d, *J* = 4 Hz, H-9), 2.68 (1H, m, H-11), 2.35 (1H, m, H-7), 1.70 (1H, d, *J* = 2.8 Hz), 1.65, 1.52 (4H, m, H-2, 3), 1.52 (3H, s, H-15), 1.19 (3H, s, H-14), 1.17 (3H, d, *J* = 7.2 Hz, H-13); ^13^C NMR (100 MHz, Methanol-d_4_) *δ*: 183.7 (C-12), 76.9 (C-1), 73.4 (C-8), 72.5 (C-9), 72.1 (C-4), 66.8 (C-6), 54.8 (C-7), 44.4 (C-5), 43.5 (C-10), 38.8 (C-3), 28.8 (C-2), 26.1 (C-14), 15.3 (C-15), 14.1 (C-13). HRESIMS *m*/*z* 301.1645 [M+H]^+^ (calcd. for C_15_H_24_O_6_+H, 301.1646).

### 3.8. General Procedure for the Synthesis of ***2*** Hydrolysis Derivatives ***17***–***18***

The appropriate amounts of **2** (0.177 mmol) and DBU (100 ul) were dissolved in DMSO (2 mL) for the reaction. The reaction mixture was stirred at room temperature for 79 h. The pH of the mixture was adjusted to 6–7 with 10% dilute hydrochloric acid and the products **17** and **18** were purified by HPLC.

(4R,4aR,5S,8S,8aS)-8-acetoxy-5-hydroxy-3,5,8a-trimethyl-2-oxo-2,4,4a,5,6,7,8,8a-octahydronaphtho[2,3-b]furan-4-yl isobutyrate (compound **17**). Yield: 15 mg (21%), purity 95%. C_21_H_28_O_7_, white amorphous powder. ^1^H NMR (400 MHz, CDCl_3_) δ: 6.64 (1H, d, J = 2.8 Hz, H-6), 5.72 (1H, s, H-9), 4.61 (1H, m, H-1), 2.56 (1H, m, H-2″), 2.13 (3H, s, H-2′), 2.00, 1.66 (2H, m, H-2), 1.95 (3H, s, H-13), 1.86 (1H, d, J = 2.8 Hz, H-5), 1.80, 1.64 (2H, m, H-3), 1.42 (3H, s, H-15), 1.35 (3H, s, H-14), 1.17 (6H, d, J = 6.8 Hz, H-3″, 4″); ^13^C NMR (100 MHz, CDCl_3_) δ: 176.7 (C-1″), 170.7 (C-12), 170.6 (C-1′), 147.5 (C-8), 144.0 (C-7), 125.1 (C-11), 115.4 (C-9), 77.3 (C-1), 70.6 (C-4), 61.8 (C-6), 53.6 (C-5), 41.3 (C-3), 40.8 (C-10), 34.6 (C-2′′), 24.9 (C-14), 24.8 (C-2), 21.3 (C-2′), 19.1 (C-3″), 18.7 (C-4″), 18.0 (C-15), 9.4 (C-13). HRESIMS *m*/*z* 393.1902 [M+H]^+^ (calcd. for C_21_H_29_O_7_, 393.1907).

(4R,4aR,5S,8S,8aS)-4,5-dihydroxy-3,5,8a-trimethyl-2-oxo-2,4,4a,5,6,7,8,8a-octahydronaphtho[2,3-b]furan-8-yl acetate (compound **18**). Yield: 3.1 mg (6%), purity 92%. C_17_H_22_O_6_, white amorphous powder. ^1^H NMR (400 MHz, CDCl_3_) δ: 6.71 (1H, d, J = 2.9 Hz, H-6), 6.12 (1H, s, -OH), 5.73 (1H, s, H-9), 5.68 (1H, t, J = 1.5 Hz, H-1), 4.64 (1H, s), 2.13 (3H, s, H-13), 2.00 (3H, s, H-1′), 2.03, 1.82 (4H, m, H-2, 3), 1.90 (1H, d, J = 2.9 Hz, H-5), 1.45 (3H, s, H-15), 1.35 (3H, s, H-14); ^13^C NMR (100 MHz, CDCl_3_) δ: 170.4 (C-1′), 166.9 (C-12), 143.5 (C-8), 127.4 (C-7), 125.6 (C-11), 115.0 (C-9), 77.2 (C-1), 70.6 (C-4), 62.0 (C-6), 53.6 (C-5), 41.3 (C-3), 40.6 (C-10), 38.9, 24.7 (C-14), 21.2(C-2′), 18.5 (C-2), 18.0 (C-15), 9.4 (C-13). HRESIMS *m*/*z* 321.1344 [M-H]^−^ (calcd. for C_17_H_21_O_6_, 321.1333).

### 3.9. X-ray Crystallographic Analysis

X-ray data were collected on an Agilent Technologies Gemini A Ultra system diffractometer with Cu-Kα radiation (*λ* = 1.5418 Å). The structure was solved by direct methods (SHELXS-97) and refined using full-matrix least-squares difference Fourier techniques. Crystallographic data (excluding structure factors) for **4**, **6**, and **14** have been deposited with the Cambridge Crystallographic Data Centre: CCDC reference numbers 2304356, 2304357, and 2304358, respectively. These data can be obtained free of charge from the Cambridge Crystallographic Data Centre via http://www.ccdc.cam.ac.uk/data_request/cif (accessed on 30 October 2023).

X-ray crystal data for **4**: C_19_H_24_O_7_, *f*_w_ = 728.76, 293 (2) K, *a* = 9.8333(2) Å, *b* = 16.4144(2) Å, *c* = 12.0115(3) Å, *α* = 90°, *β* = 103.202(2)°, *γ* = 90°, *V* = 1887.51(7) Å^3^, space group P2_1_, Z = 2, *μ* (MoK_α_) = 0.816 mm^−1^, *F*(000) = 776.0, *ρ*_calc_ = 1.282 g/cm^3^; 22,829 reflections measured, 6707 were unique (R_int_ = 0.0399); 2Θ range for data collection: 7.56 to 133.94°. Flack 0.08(12). The final R_1_ = 0.0352, wR_2_ = 0.0785 (I Page: 16 ≥ 2σ (I)).

X-ray crystal data for **6**: C_23_H_34_O9, *f*_w_ = 454.50, 293 (2) K, orthorhombic, *a* = 10.5090 (2) Å, *b* = 12.2547 (2) Å, *c* = 18.1235 (3) Å, *α* = 90°, *β* = 90°, *γ* = 90°, *V* = 2,334.03 (7) Å^3^, space group P2_1_2_1_2_1_, Z = 4, *μ* (MoK_α_) = 0.827 mm^−1^, *F*(000) = 976.0, *ρ*_calc_ = 1.293 g/cm^3^; 14,042 reflections measured, 4127 were unique (R_int_ = 0.0421); 2Θ range for data collection: 8.72 to 133.8°. Flack −0.15(18). The final R_1_ = 0.0358, wR_2_ = 0.1048 (I Page: 16 ≥ 2σ (I)).

X-ray crystal data for **14**: C_19_H_30_O_7_, *f*_w_ = 370.20, 293 (2) K, monoclinic, *a* = 8.6601 (6) Å, *b* = 13.3921 (8) Å, *c* = 9.3091 (7) Å, *α* = 90°, *β* = 112.750 (9)°, *γ* = 90°, *V* = 995.65 (13) Å^3^, space group P2_1_, Z = 2, *μ* (MoK_α_) = 0.756 mm^−1^, *F*(000) = 378.0, *ρ*_calc_ = 1.179 g/cm^3^; 9881 reflections measured, 3473 were unique (R_int_ = 0.0674); 2Θ range for data collection: 10.304 to 135.168°. Flack −0.2(3). The final R_1_ = 0.0921, wR_2_ = 0.2422 (I Page: 16 ≥ 2σ (I)).

### 3.10. Biological Evaluation

#### 3.10.1. Cell Lines and Cell Culture

Huh7 and HepG2 cells were obtained from the Shanghai Institute of Biochemistry and Cell Biology, Chinese Academy of Sciences. Both cancer cells were grown in MEM medium, containing 10% fetal bovine serum (FBS) and 1% penicillin–streptomycin, and incubated at 37 °C in a humidified atmosphere containing 5% CO_2_.

#### 3.10.2. Cell Cytotoxicity Assay

Compound-induced cytotoxicity was analyzed in Huh7 and HepG2 cells using CCK-8 assays. Generally, the cells were cultured in MEM supplemented with 10% FBS in 5% CO_2_ at 37 °C. Cells were seeded in 96-well plates at a density of 8000 cells/well. The media were replaced on the next day. Afterward, the cultures were incubated with compound **10** at 37 °C for 24 h, a CCK-8 assay was performed to examine cell viability, and the absorbance was measured at 570 nm.

#### 3.10.3. Calcein-AM/PI Costaining

Huh7 and HepG2 cells were seeded in 6-well cell culture plates. The cells were then exposed to various doses of **10** for 24 h. The cells were rinsed three times with PBS, trypsinized, and centrifuged, and the defined amount of them was resuspended in 100 µL 1× assay buffer, in which live and dead cells were then costained by a mixture of Calcein-AM (2 mM) and PI (1.5 mM) solution for 20 min. Afterward, fluorescence microscopic images of cells were taken using an inverted fluorescent microscope (OLYMPUS IX-51, OLYMPUS, Tokyo, Japan).

#### 3.10.4. Colony Formation Assay

Huh7 and HepG2 cells were seeded in 6-well cell culture plates (1000 cells per well). After a 24 h incubation period, different concentrations of **10** were added to the cell. The cells were cultured for 14 d in the incubator, with the media being changed every 3 d. The cells were then fixed for 30 min with 4% paraformaldehyde and stained for 30 min with 0.1% crystal violet. After washing, the plates were photographed and counted using ImageJ2×.2.1.5.0 software. The clone formation rate relative to the solvent control group was calculated.

#### 3.10.5. Cell Apoptosis Analysis

Huh7 and HepG2 cells were seeded in 6-well cell culture plates. The cells were then grown for 24 h with varied doses of **10**, and subsequent processes were carried out based on the explanatory memorandum of the Annexin V-FITC/PI Apoptosis Detection Kit (Yeasen, Cat.#: 40302ES60, Shanghai, China). Briefly, cells were digested by trypsin, centrifuged at 4 °C with 1500 rpm for 5 min, washed with pre-cooling PBS twice, and resuspended in 100 μL 1 × binding buffer before staining with 5 μL Annexin-V-FITC and 10 μL PI staining solution for 10–15 min incubation in the darkness. After diluting with 400 μL of 1 × binding buffer, the samples were examined by flow cytometry (Sysmex-Partec CyFlow^TM^ Cube 6). Data were analyzed using FlowJo V1 (Flexera Software, Chicago, IL, USA).

#### 3.10.6. Cell Cycle Arrest Analysis

Huh7 and HepG2 cells were seeded in 6-well cell culture plates. The cells were then exposed to various doses of **10** for 24 h. The cells were harvested, washed three times with PBS, and then fixed for at least 12 h in cold 70% ethanol at 4 °C. After that, all of the cells were washed three times in PBS to remove any remaining ethanol. The cells were then resuspended in the solution stained with propidium iodide and incubated at room temperature for 30 min in the dark. A NovoCyte Flow Cytometer (Agilent, Santa Clara, CA, USA) was used to analyze the samples.

#### 3.10.7. Wound Healing Assay

Cells (1 × 10^6^/mL) were seeded in 6-well plates and cultured until each well was covered. The cell monolayer was scraped vertically with a 200 μL sterile pipette tip and rinsed 3 times with sterile PBS. After that, 5% FBS medium was added with the compounds to be tested and incubated for 48 h. Images were taken at 0 and 48 h for each scratch by microscope (OLYMPUS IX-51). ImageJ software was used to compare the edge-by-edge measurements at 0 h and 24 h.

#### 3.10.8. Cell Migration Assays

Cell migration assays were performed in the chamber of a 24-well Transwell plate. For migration experiments, starved cells were suspended in a medium without serum with a density of 1 × 10^5^/mL. 200 μL of prepared cell suspension; various concentrations of compounds were added to the upper chamber of the chamber and 600 μL of medium (20% FBS) was added to the outer chamber of the chamber. The plates were incubated in an incubator at 37 °C for 48 h. Then, the cells in the upper chamber were swabbed, and the rest of the cells in the chamber were fixed with 4% paraformaldehyde for 30 min, stained with 0.1% crystal violet, and photographed (OLYMPUS IX-51) for observation.

#### 3.10.9. ADME Prediction

Computational ADME analysis was performed using SwissADME (http://www.swissadme.ch/, accessed on 14 September 2023) to estimate the physiochemical, lipophilicity, water-solubility, pharmacokinetics, drug-likeness, and medicinal chemistry properties of the compound.

#### 3.10.10. Statistical Analysis

All statistical analyses were performed using GraphPad-Prism software 9. All data are expressed as the mean ± SD for three independent tests. The statistical significance of the data between groups was acquired by either Student’s test or one-way ANOVA multiple comparisons.

## 4. Conclusions

In this study, a new series of SL derivatives (**3**–**18**) were synthesized using epimers **1** and **2** as the parent molecules, isolated from *Sphagneticola trilobata*, and evaluated for their anti-HCC activity. Furthermore, the structures of **4**, **6**, and **14** were confirmed by X-ray single-crystal diffraction analyses. The cytotoxic activities of **3**–**18** on two HCC cell lines, including HepG2 and Huh7, were evaluated using the CCK-8 assay. Among them, compound **10** exhibited the best activity against HepG2 and Huh7 cell lines with IC_50_ values of 9.73 and 18.86 µM, respectively. It was suggested that the double bonds at the C-4, 14 or C-3, 4 positions were more favorable to enhancing the activity of HCC. Notably, the double bond between C-11 and C-13 was reduced to the methyl group in all the compounds, which were less potent than the parental compounds, further confirming that SLs’ cytotoxic activity is mainly dependent on the *α*-methylene-*γ*-lactone group. Further studies showed that **10** induced cell apoptosis, arrested the cell cycle in the S phase, and induced the inhibition of cell proliferation and migration in HepG2 and Huh7. ADME properties prediction showed that **10** may possess the properties to be a drug candidate. Thus, **10** may be a promising lead compound for the treatment of HCC.

## Figures and Tables

**Table 1:** Cytotoxic activity of compounds **1**–**18** in HepG2 and Huh7 cells.

Compound	IC_50_ (μM)				
	HepG2	Huh7	Compound	HepG2	Huh7
**1**	27.77 ± 0.92	28.15 ± 0.87	**11**	47.98 ± 2.53	56.69 ± 1.09
**2**	22.12 ± 1.13	14.04 ± 2.37	**12**	>70	>70
**3**	14.42 ± 1.69	24.17 ± 0.57	**13**	>70	>70
**4**	39.43 ± 2.84	44.07 ± 3.30	**14**	>70	>70
**5**	15.00 ± 1.07	49.69 ± 2.46	**15**	>70	>70
**6**	>70	>70	**16**	>70	>70
**7**	>70	>70	**17**	>70	>70
**8**	>70	>70	**18**	>70	>70
**9**	>70	>70	adriamycin	26.80 ± 1.33	23.87 ± 1.62
**10**	9.73 ± 1.72	18.86 ± 0.88			

IC_50_: concentration of the compound that is required for 50% inhibition of cell growth.

**Table 2:** Physiochemical, lipophilicity, water-solubility, pharmacokinetics, drug-likeness, and medicinal chemistry properties of compound **10**.

Physicochemical Properties		Water Solubility	
Formula	C_21_H_28_O_7_	Log S (ESOL)	−3.35
Molecular weight	392.44 g/mol	Solubility	1.76 × 10^−1^ mg/mL; 4.49 × 10^−4^ mol/L
Num. heavy atoms	28	Class	Soluble
Num. arom. heavy atoms	0	Log S (Ali)	−3.95
Fraction Csp3	0.67	Solubility	4.43 × 10^−2^ mg/mL; 1.13 × 10^−4^ mol/L
Num. rotatable bonds	5	Class	Soluble
Num. H-bond acceptors	7	Log S (SILICOS-IT)	−2.30
Num. H-bond donors	1	Solubility	1.99 × 10^0^ mg/mL; 5.06 × 10^−3^ mol/L
Molar Refractivity	100.53	Class	Soluble
TPSA	99.13 Å^2^	Pharmacokinetics	
Lipophilicity		GI absorption	High
Log Po/w (iLOGP)	2.71	BBB permeant	No
Log Po/w (XLOGP3)	2.23	P-gp substrate	No
Log Po/w (WLOGP)	1.93	CYP1A2 inhibitor	No
Log Po/w (MLOGP)	2.04	CYP2C19 inhibitor	No
Log Po/w (SILICOS-IT)	2.12	CYP2C9 inhibitor	No
Consensus Log Po/w	2.20	CYP2D6 inhibitor	No
Druglikeness		CYP3A4 inhibitor	No
Lipinski	Yes; 0 violation	Log Kp (skin permeation)	−7.11 cm/s
Ghose	Yes	Medicinal Chemistry	
Veber	Yes	PAINS	0 alert
Egan	Yes	Brenk	3 alerts: isolated_alkene, michael_acceptor_1, more_than_2_esters
Muegge	Yes	Lead-likeness	No; 1 violation: MW > 350
Bioavailability Score	0.55	Synthetic accessibility	5.32

## Data Availability

The data presented in this study are available in Supplementary Materials.

## Supplementary Materials

The following supporting information can be downloaded at: https://www.mdpi.com/article/10.3390/molecules29020393/s1, The preparation process of **1** and **2**; ^1^H NMR, ^13^C NMR, and HRESIMS of compounds **3**–**18** (Figures S1–S55) (PDF).
