# Supplementary material for: Design, Synthesis, and Anti-Hepatocellular Carcinoma Evaluation of Sesquiterpene Lactone Epimers Trilobolide-6-O-isobutyrate Analogs
Source: Molecules. 2024 Jan 12;29(2):393. doi: 10.3390/molecules29020393 (PMC10821474; doi:10.3390/molecules29020393)
Supplement: Supplementary file 1 [file molecules-29-00393-s001.zip › molecules-2798667-supplementary.pdf]

SUPPLEMENTARY MATERIALS

Design, Synthesis, and Anti-Hepatocellular Carcinoma Evaluation of Sesquiterpene Lactone Epimers Trilobolide-6-*O*-isobutyrate Analogs

Xiuqiao Zhou [1,2,†], Guohui Yi [3,†], Yiming Qian [1,2], Xiaorong Yang [1,2], Guangying Chen [1,2], Yang Hui [1,2] and Wenhao Chen [1,2,*]

[1] Key Laboratory of Tropical Medicinal Resource Chemistry of Ministry of Education, College of Chemistry and Chemical Engineering, Hainan Normal University, Haikou 571158, China
[2] Key Laboratory of Tropical Medicinal Plant Chemistry of Hainan Province, College of Chemistry and Chemical Engineering, Hainan Normal University, Haikou 571158, China
[3] Public Research Center, Hainan Medical University, Haikou 571199, China; guohuiyi6@hainmc.edu.cn
* Correspondence: 070103@hainnu.edu.cn
† These authors contributed equally to this work.

Abstract: Hepatocellular carcinoma (HCC), one of the most common malignant cancers with a low 5-year survival rate, is the third leading cause of cancer-related deaths worldwide. The finding of novel agents and strategies for the treatment of HCC is an urgent need. Sesquiterpene lactones (SLs) have attracted extensive attention because of their potent antitumor activity. In this study, a new series of SL derivatives (**3**–**18**) were synthesized using epimers **1** and **2** as parent molecules, isolated from *Sphagneticola trilobata*, and evaluated for their anti-HCC activity. Furthermore, the structures of **4**, **6**, and **14** were confirmed by X-ray single-crystal diffraction analyses. The cytotoxic activities of **3**–**18** on two HCC cell lines, including HepG2 and Huh7, were evaluated using the CCK-8 assay. Among them, compound **10** exhibited the best activity against the HepG2 and Huh7 cell lines. Further studies showed that **10** induced cell apoptosis, arrested the cell cycle at the S phase, and induced the inhibition of cell proliferation and migration in HepG2 and Huh7. In addition, absorption, distribution, metabolism, and excretion (ADME) properties prediction showed that **10** may possess the properties to be a drug candidate. Thus, **10** may be a promising lead compound for the treatment of HCC.

Keywords: sesquiterpene lactones; synthesis; anti-hepatocellular carcinoma evaluation

Content

1. The separation of SLs 1 and 2

The air-dried flowers of *S. trilobata* (24.0 kg) were powered and exhaustively extracted three times with 95% EtOH-H_2O (v/v) at room temperature. The extract was concentrated, and the residue was suspended in H_2O and then partitioned successively with petroleum ether, and EtOAc. The EtOAc-soluble fraction (1.30 kg) was subjected to silica gel CC, using a step gradient-elution technique, employing a mixture of $CHCl_3$/EtOAc (15:1–0:1, v/v) as solvents, to afford seven fractions (Fr 1, 15:1; Fr 2, 8:1; Fr 3, 5:1; Fr 4, 3:1, Fr 5, 2:1; Fr 6, 1:1; Fr 7, 0:1) according to TLC analysis. Fr 2 (180 g) was further purified by CC (SiO_2; petroleum ether/acetone, 15:1) to give Frs 2-1 and 2-2. Compounds **1** (20 g) and **2** (15 g) were obtained from Fr 2-1 (100 g) by CC (SiO_2; $CHCl_3$/acetone, 15:1).

2. Structural Characterization of the Compounds Spectrum (^{1}H NMR, ^{13}C NMR, Part of 2D NMR, HRESIMS) of the Final Compounds 3-18.

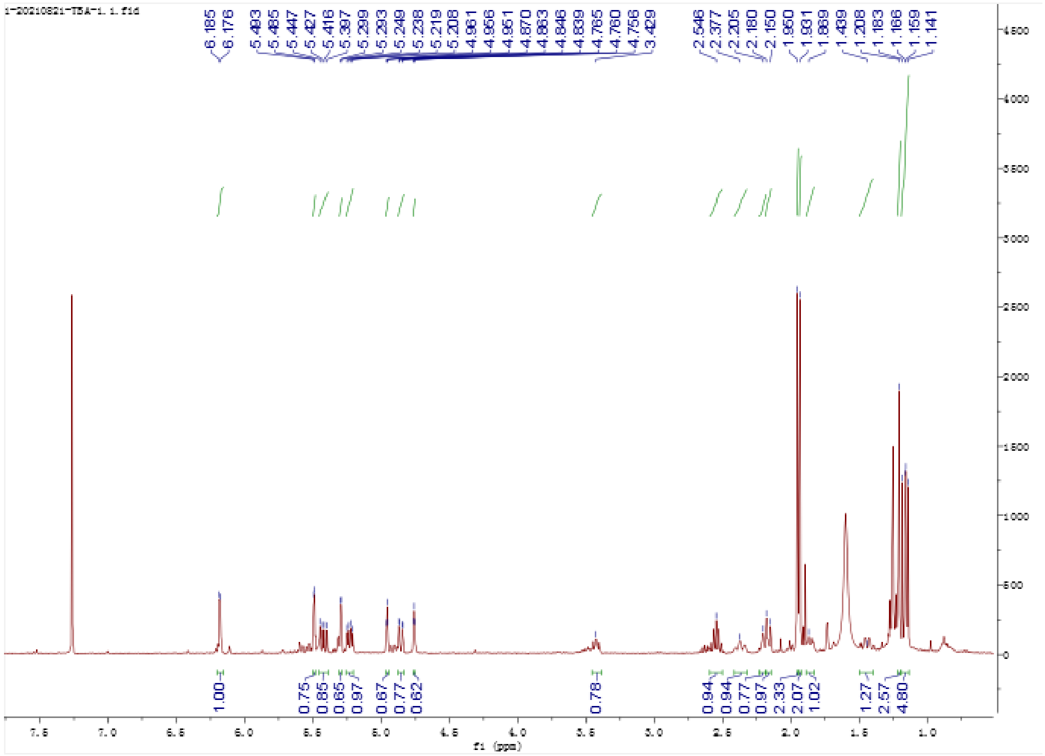

Figure S1 ^{1}H NMR spectrum of compound **3** (400 MHz in $CDCl_3$)

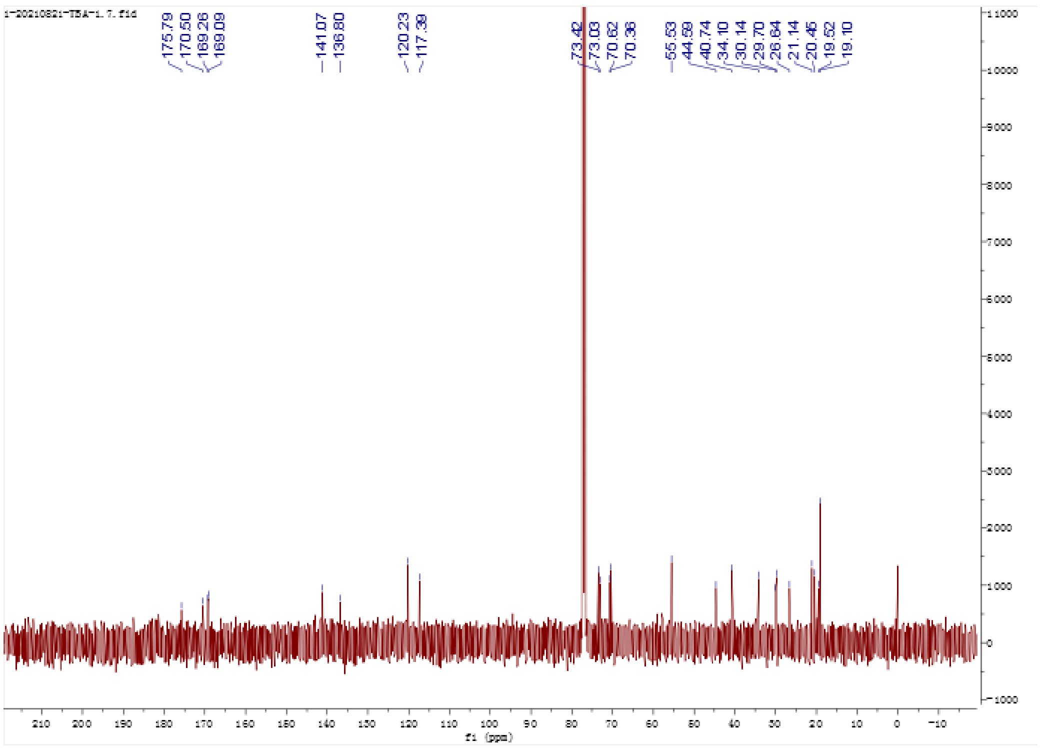

Figure S2 ^{13}C NMR spectrum of compound **3** (100 MHz in $CDCl_3$)

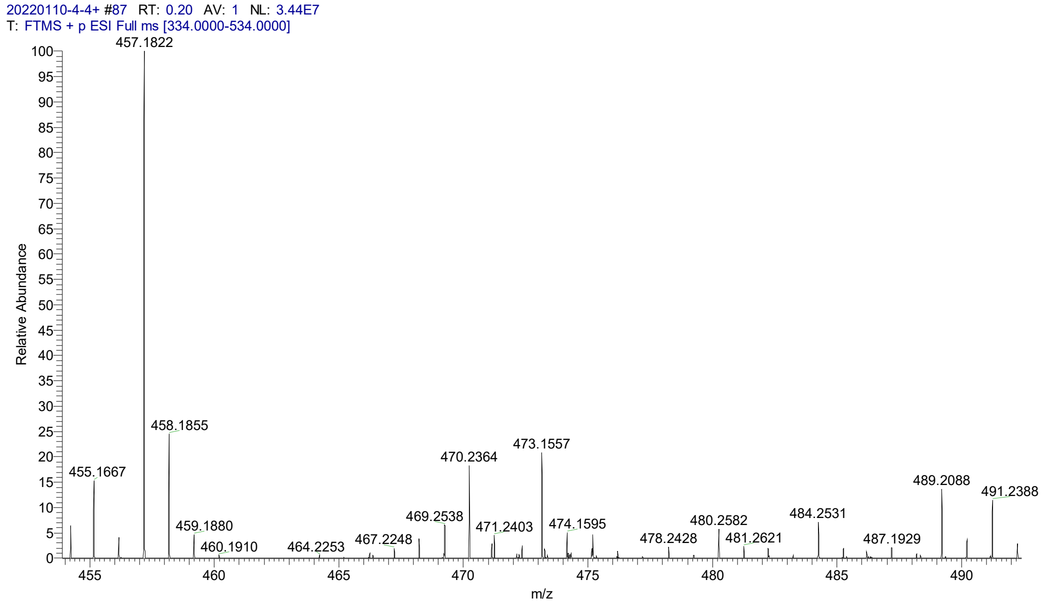

Figure S3 HRESIMS spectrum of compound **3**

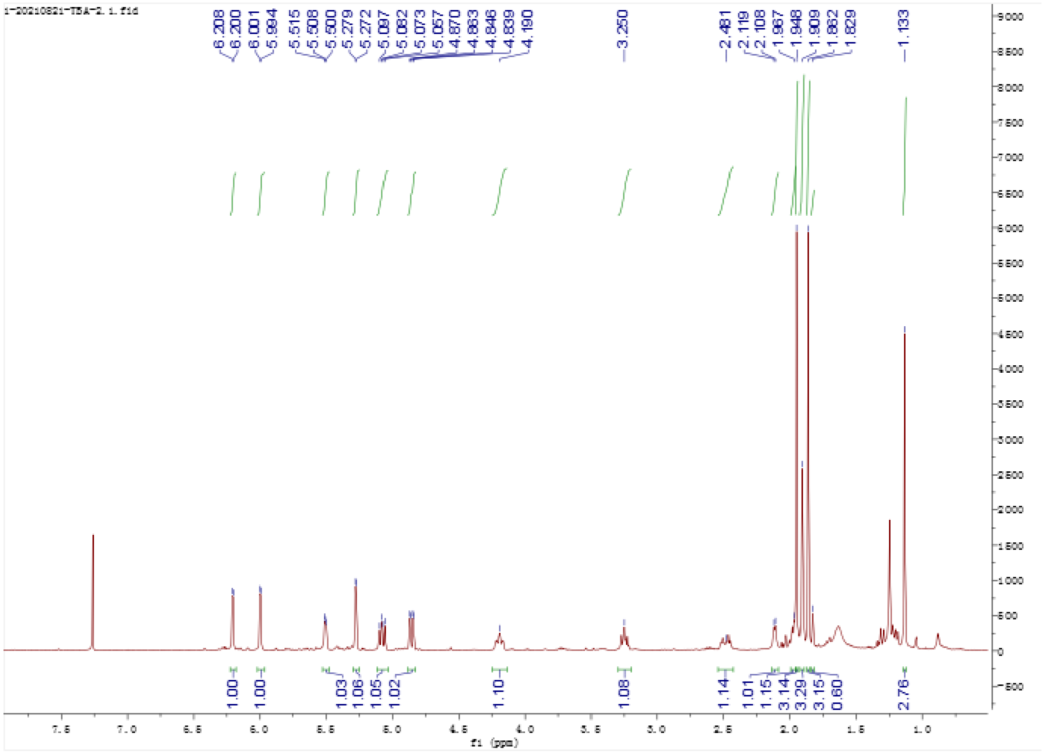

Figure S4 ^{1}H NMR spectrum of compound **4** (400 MHz in $CDCl_3$)

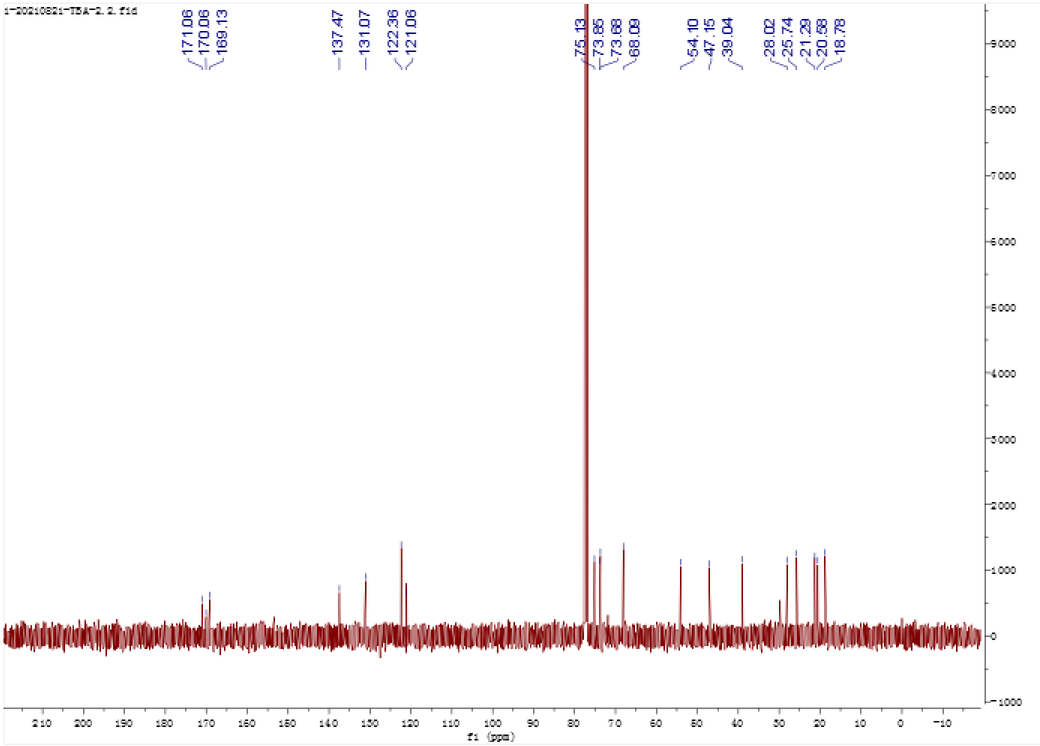

Figure S5 ^{13}C NMR spectrum of compound **4** (100 MHz in $CDCl_3$)

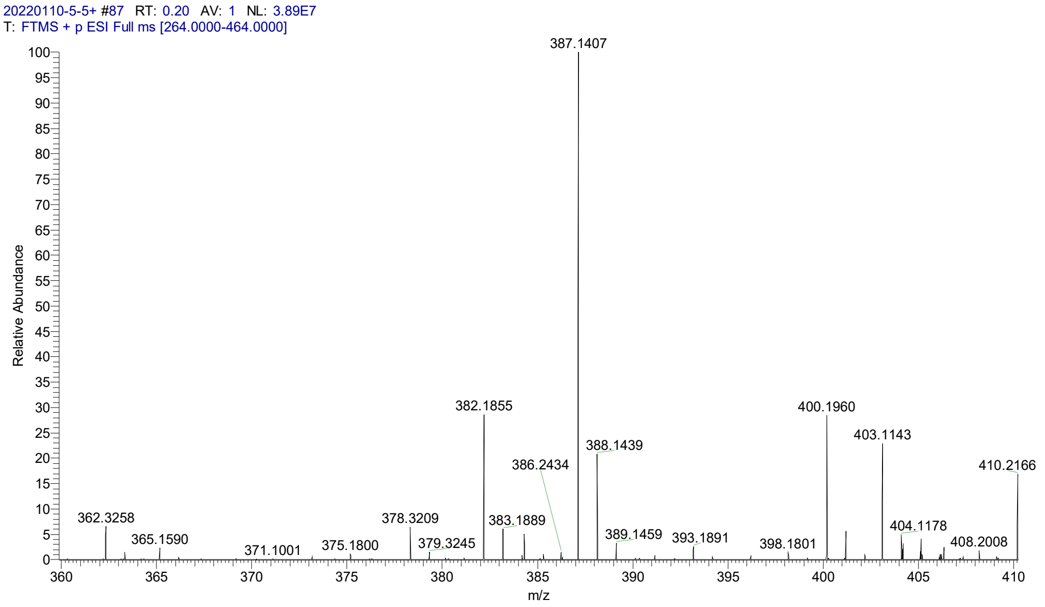

Figure S6 HRESIMS spectrum of compound **4**

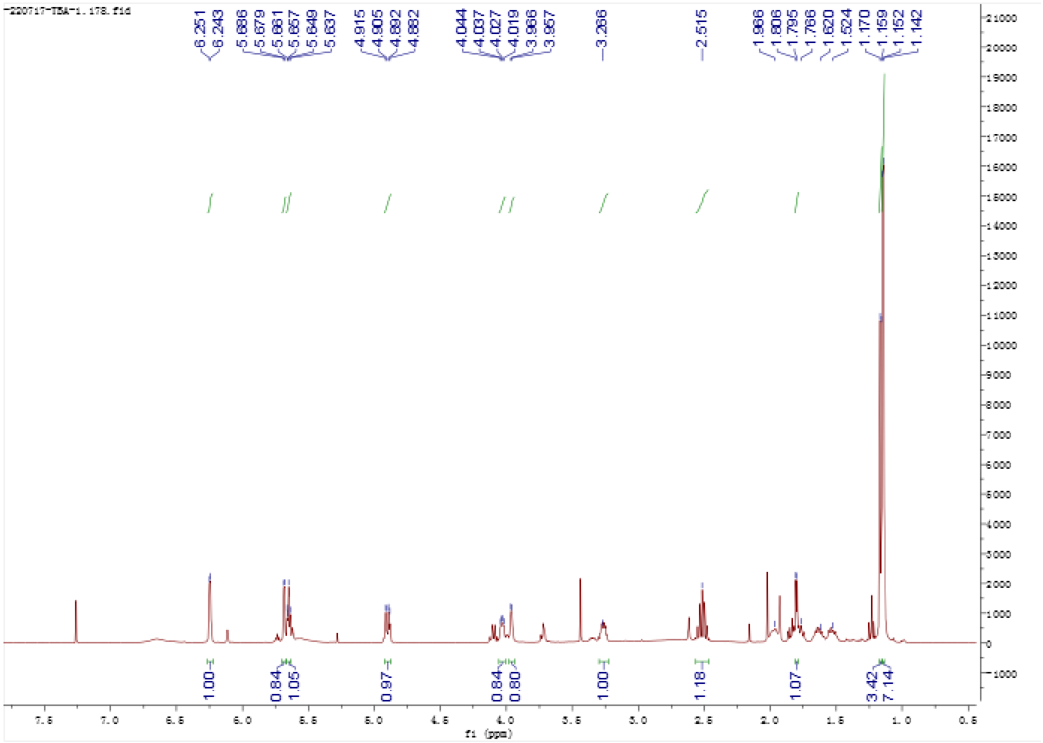

Figure S7 ^{1}H NMR spectrum of compound **5** (400 MHz in $CDCl_3$)

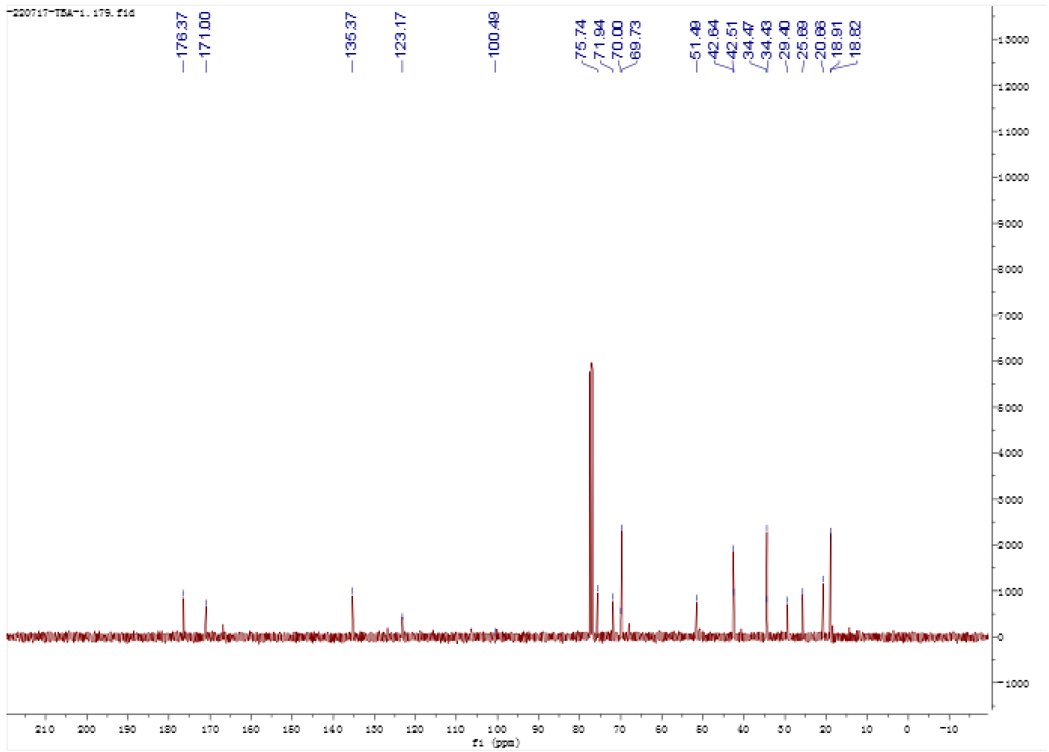

Figure S8 ^{13}C NMR spectrum of compound **5** (100 MHz in $CDCl_3$)

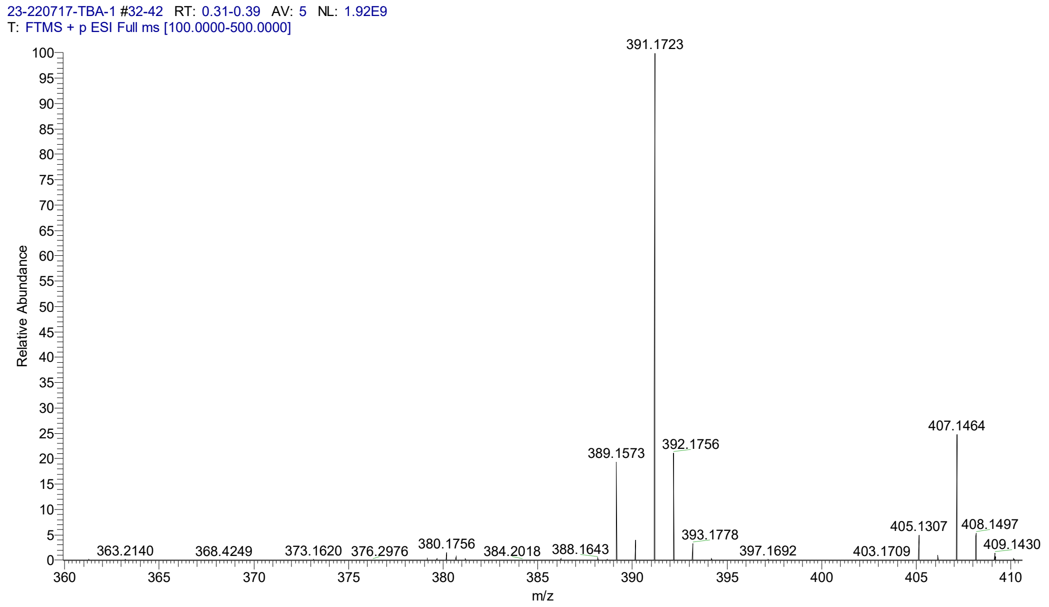

Figure S9 HRESIMS spectrum of compound **5**

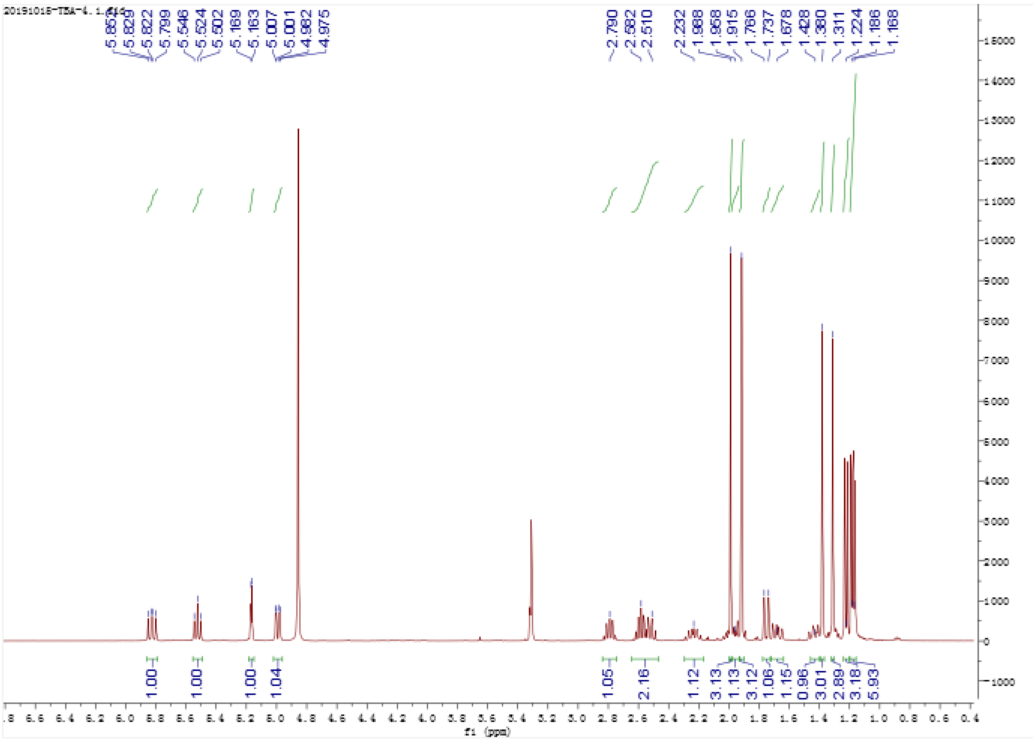

Figure S10 ^{1}H NMR spectrum of compound **6** (400 MHz in Methanol-*d*4)

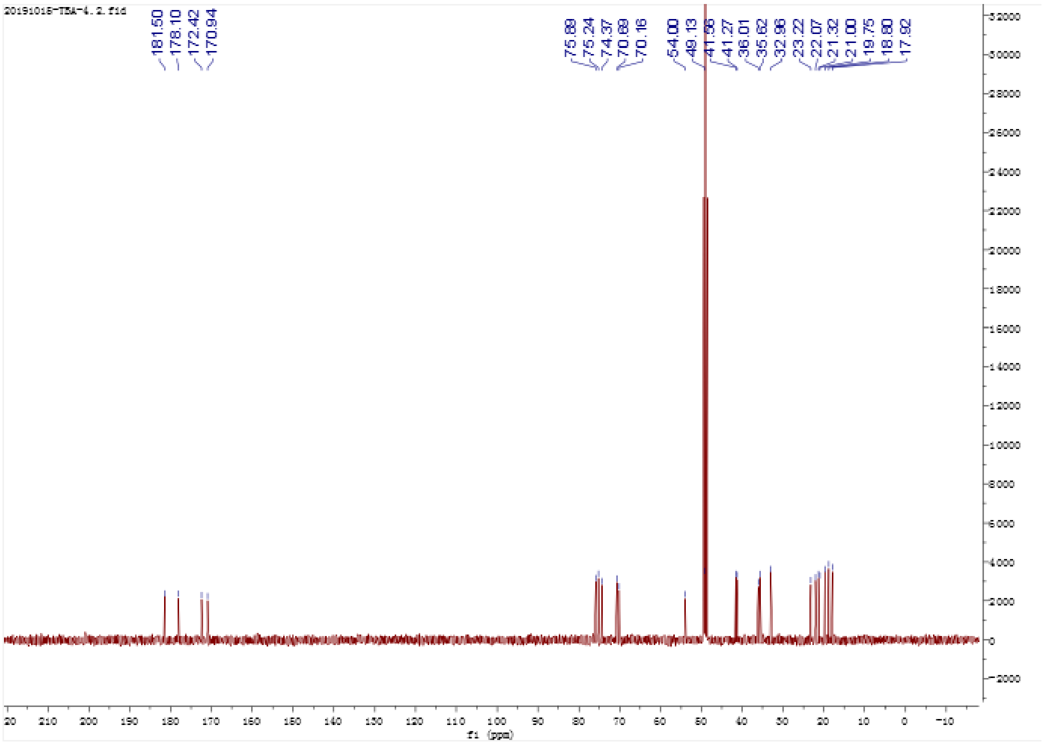

Figure S11 ^{13}C NMR spectrum of compound **6** (100 MHz in Methanol-*d*4)

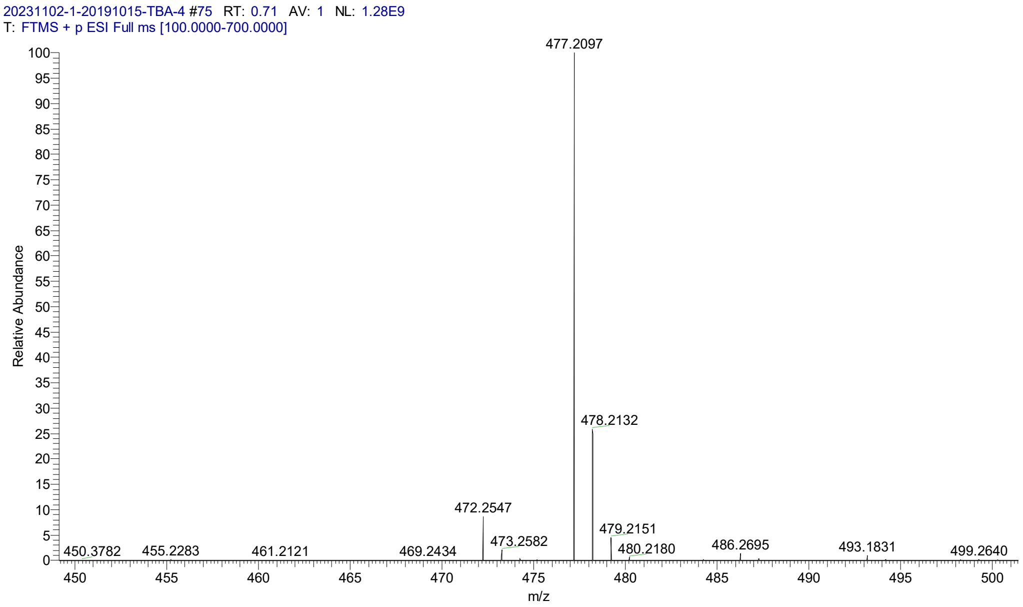

Figure S12 HRESIMS spectrum of compound **6**

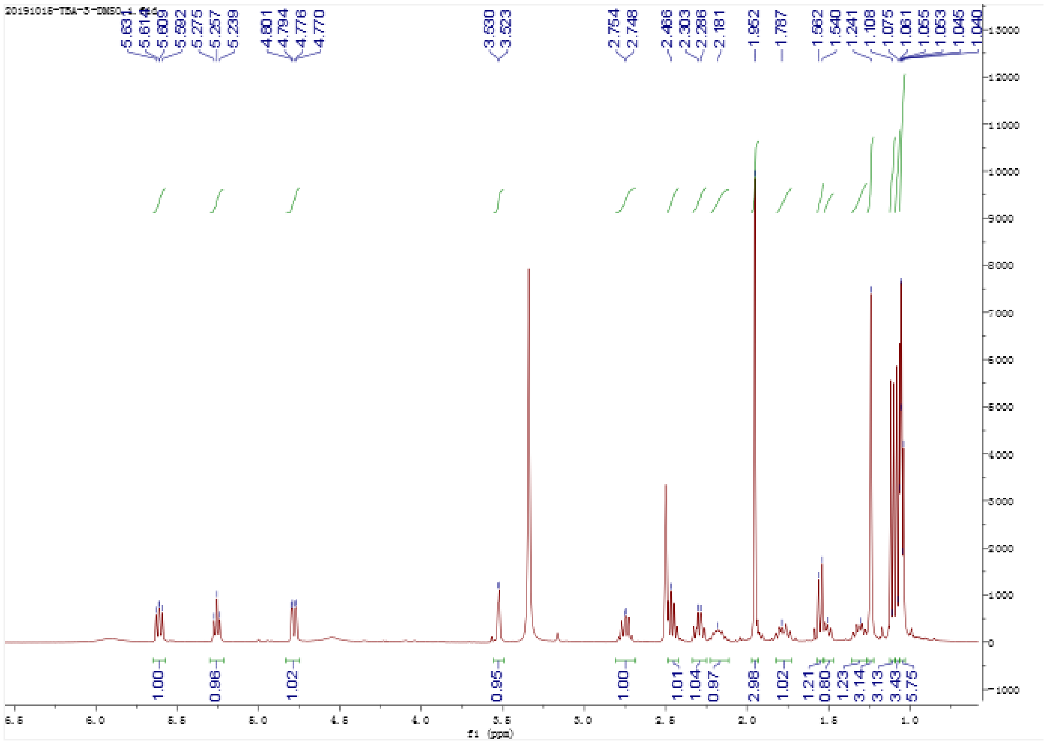

Figure S13 ^{1}H NMR spectrum of compound **7** (400 MHz in DMSO-d_6)

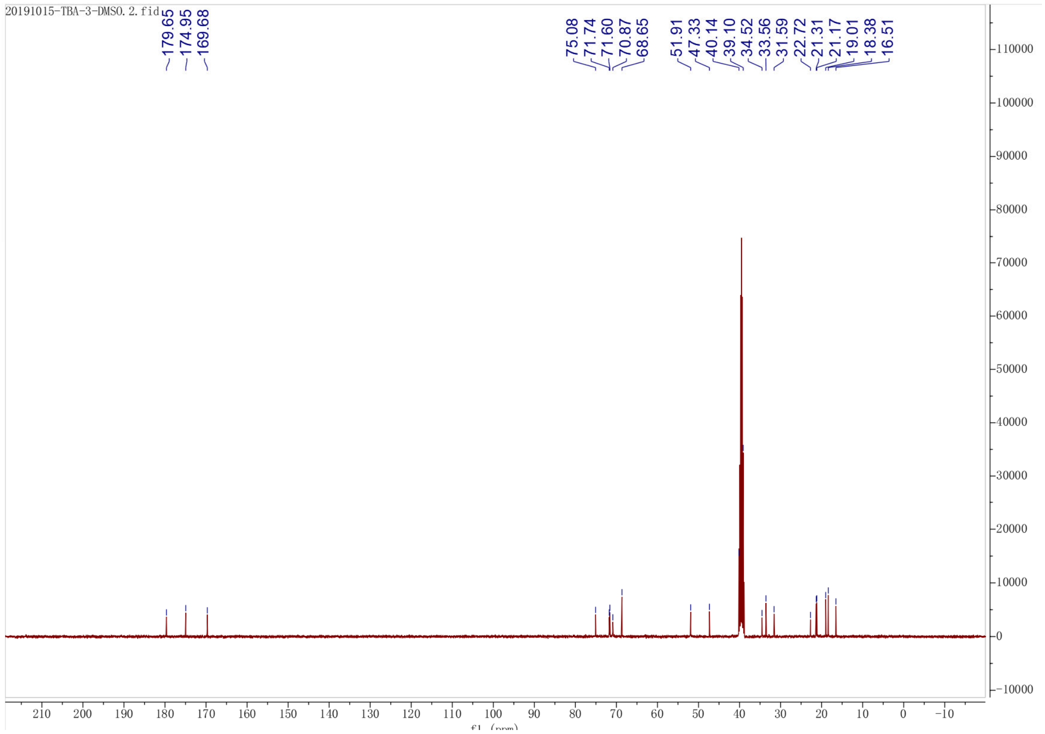

Figure S14 ^{13}C NMR spectrum of compound **7** (100 MHz in DMSO-d_6)

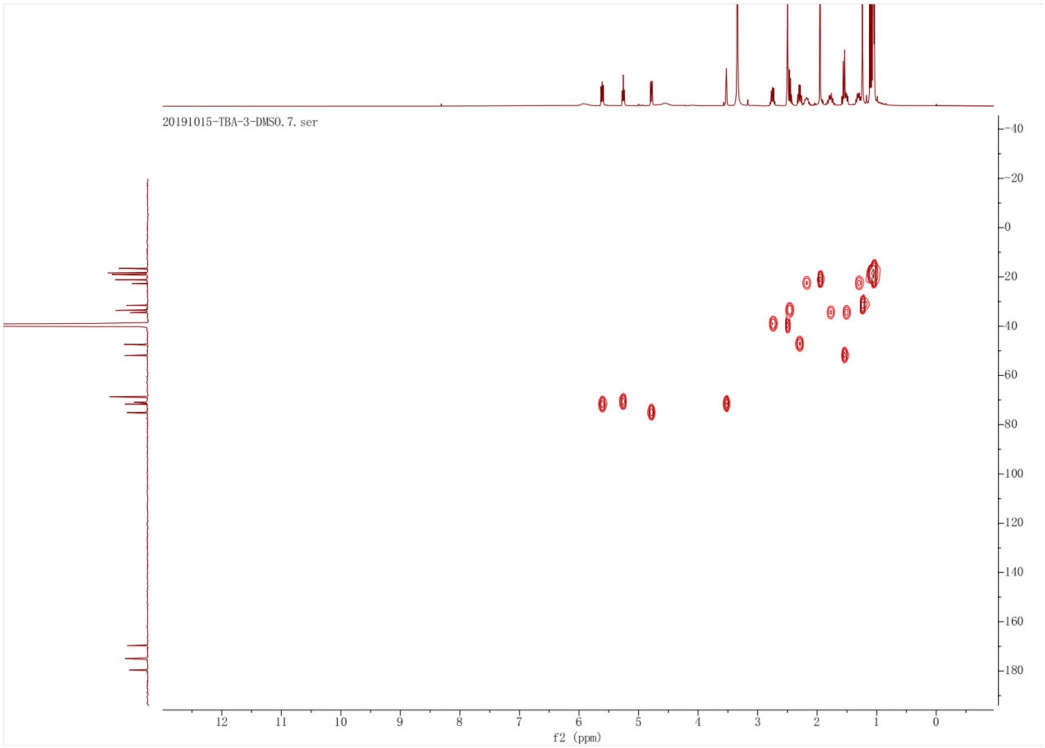

Figure S15 HSQC spectrum of **7** (100 MHz in DMSO-d_6)

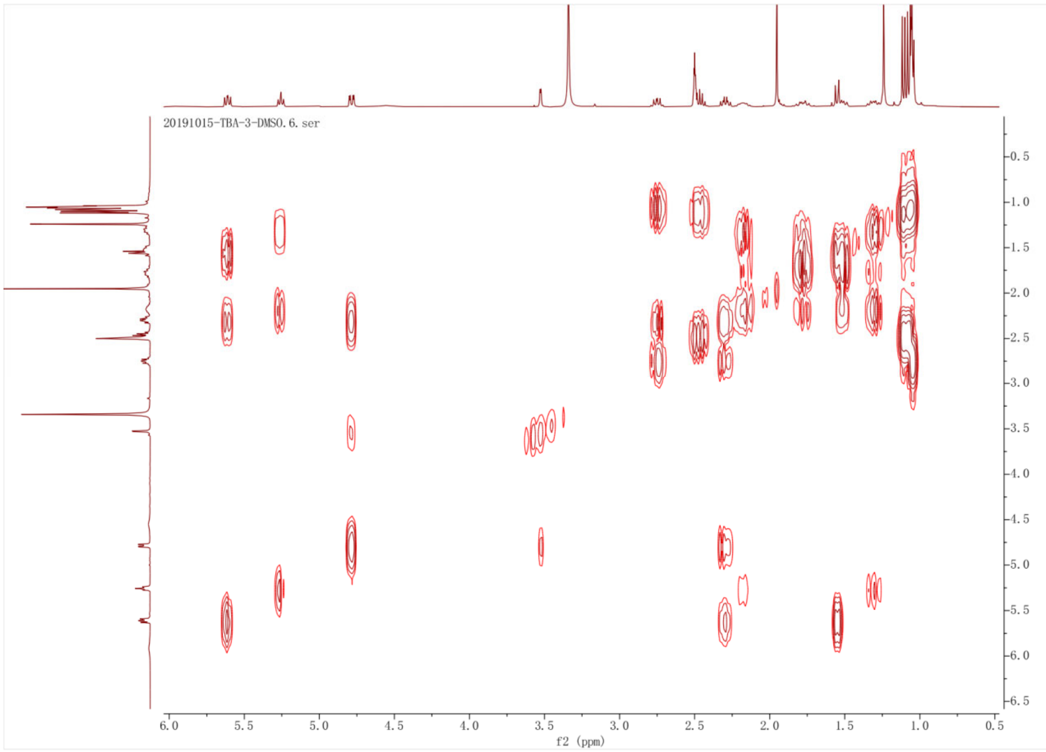

Figure S16 ^{1}H-^{1}H COSY spectrum of **7** (100 MHz in DMSO-d_6)

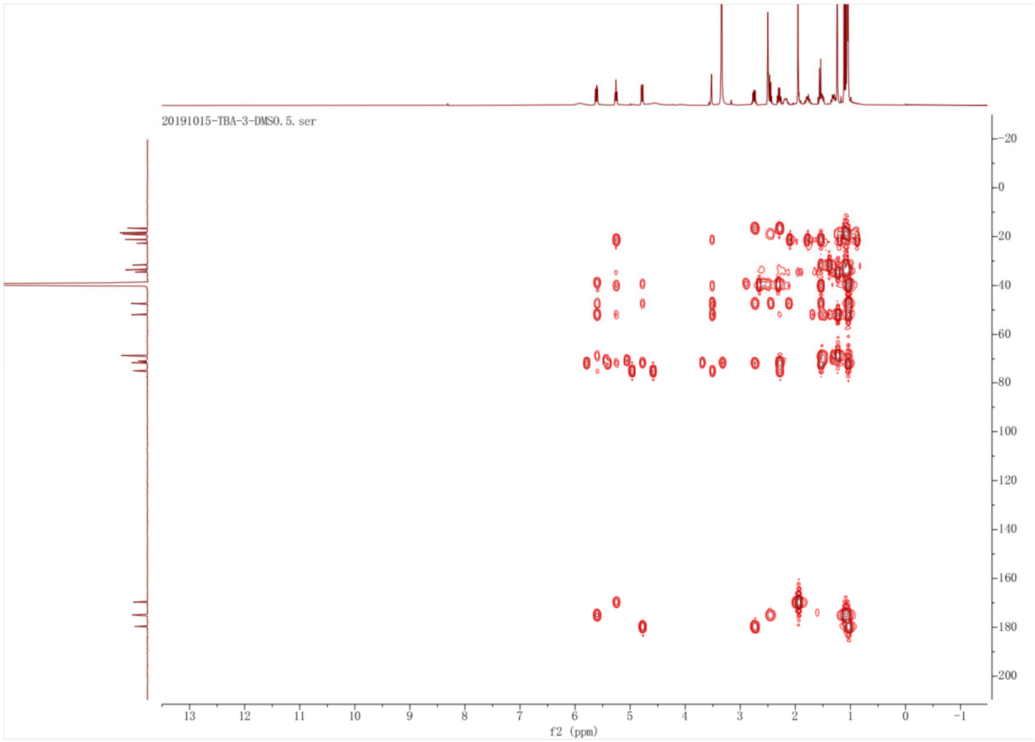

Figure S17 HMBC spectrum of **7** (100 MHz in DMSO-d_6)

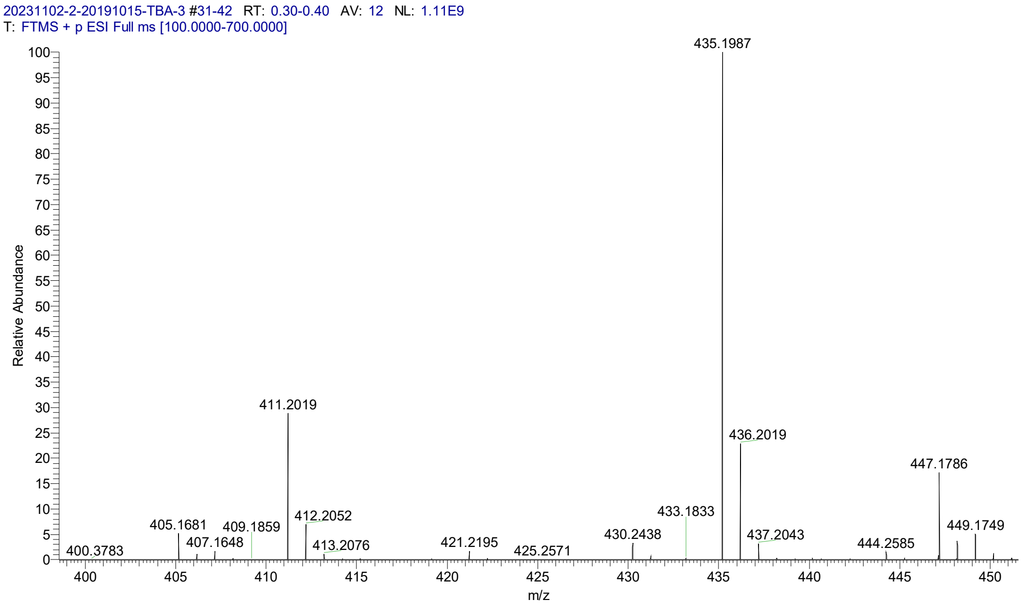

Figure S18 HRESIMS spectrum of compound **7**

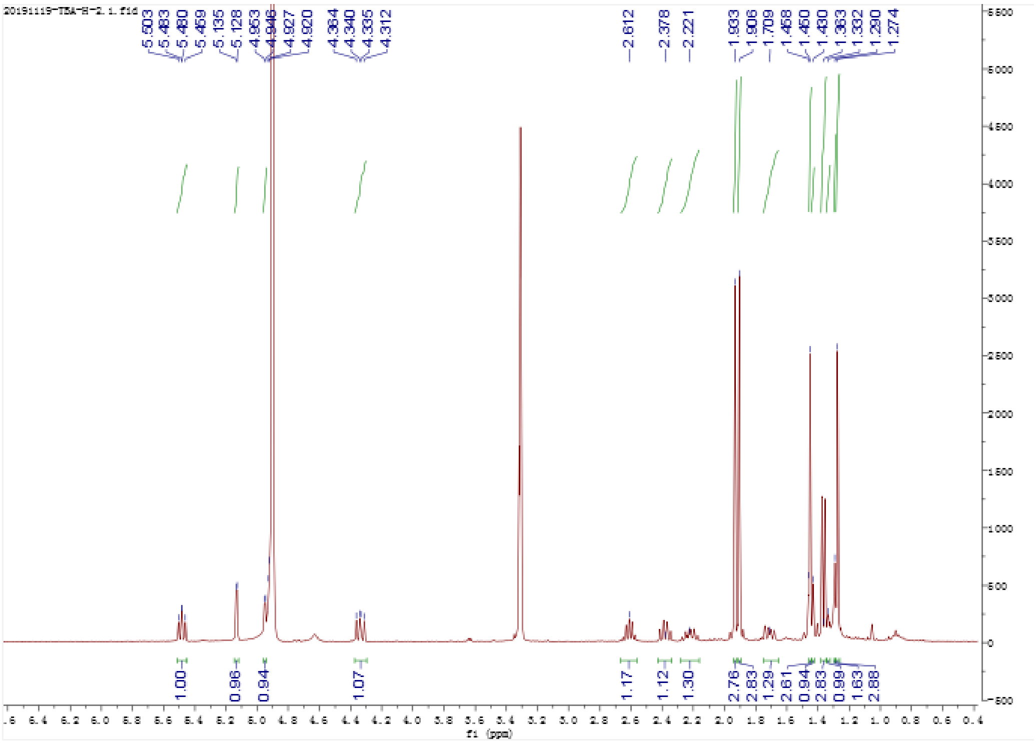

Figure S19 ^{1}H NMR spectrum of compound **8** (400 MHz in Methanol-d_4)

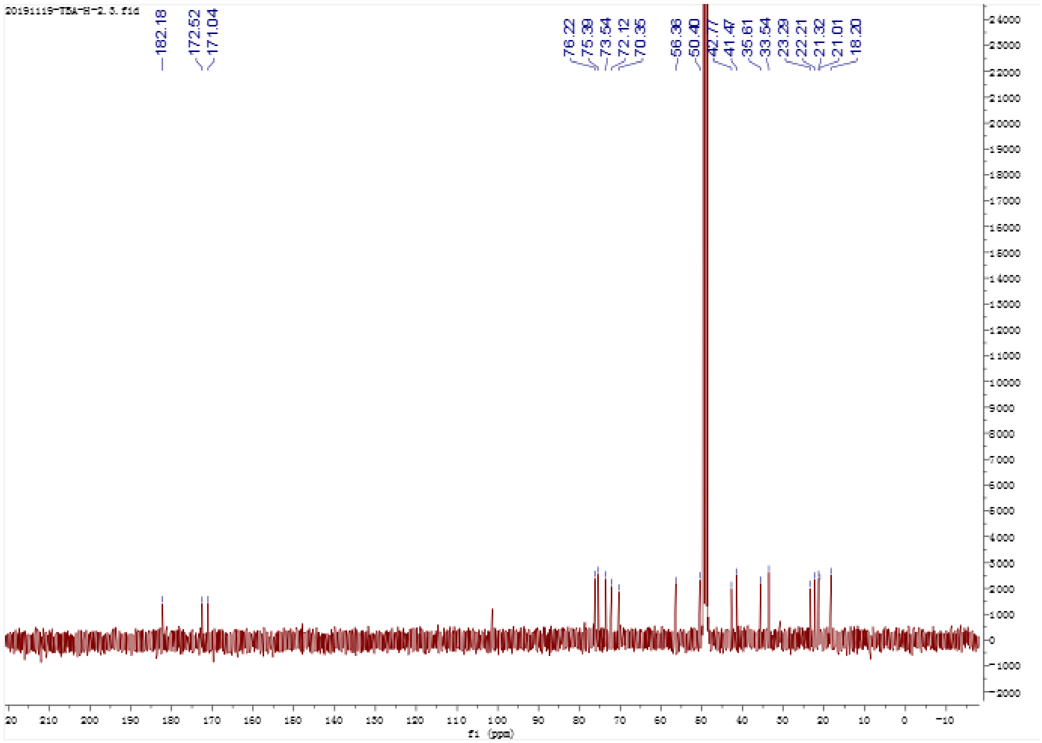

Figure S20 ^{13}C NMR spectrum of compound **8** (100 MHz in Methanol-*d*4)

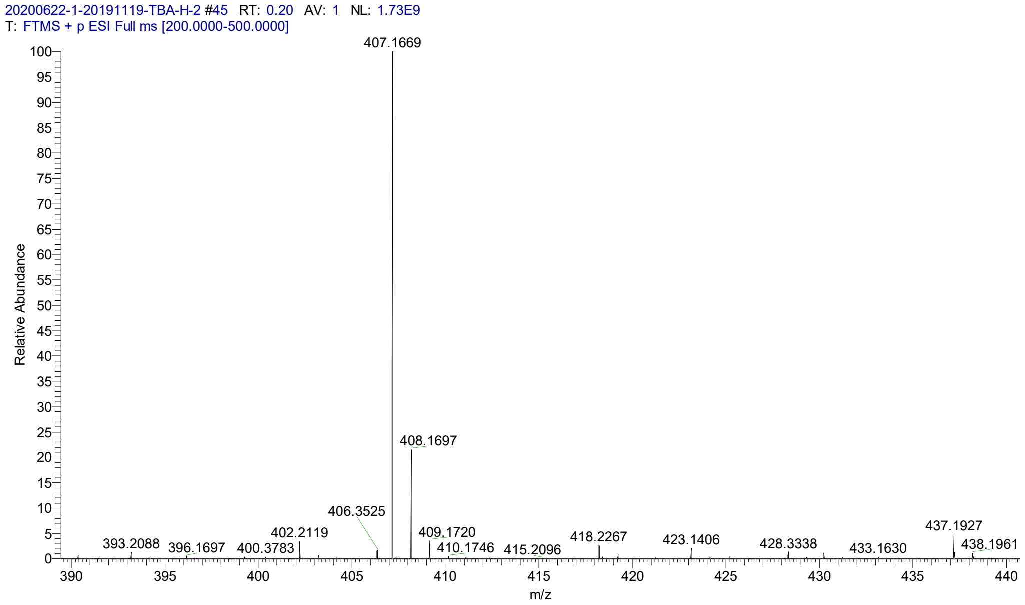

Figure S21 HRESIMS spectrum of compound **8**

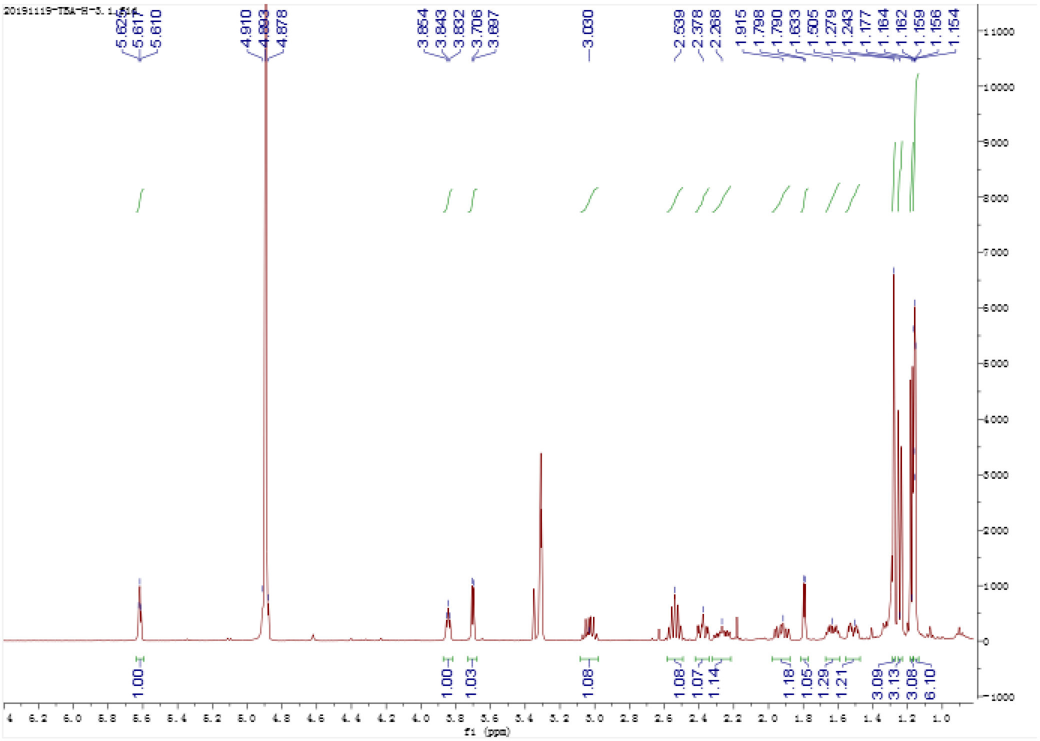

Figure S22 ^{1}H NMR spectrum of compound **9** (400 MHz in Methanol-d_4)

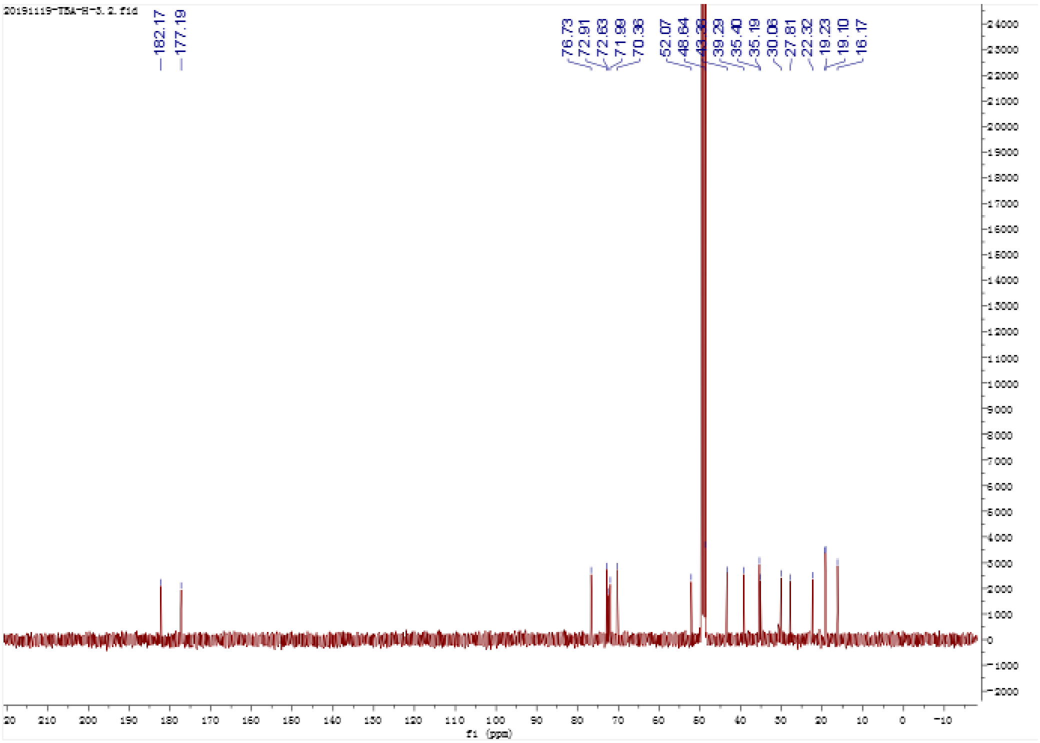

Figure S23 ^{13}C NMR spectrum of compound **9** (100 MHz in Methanol-d_4)

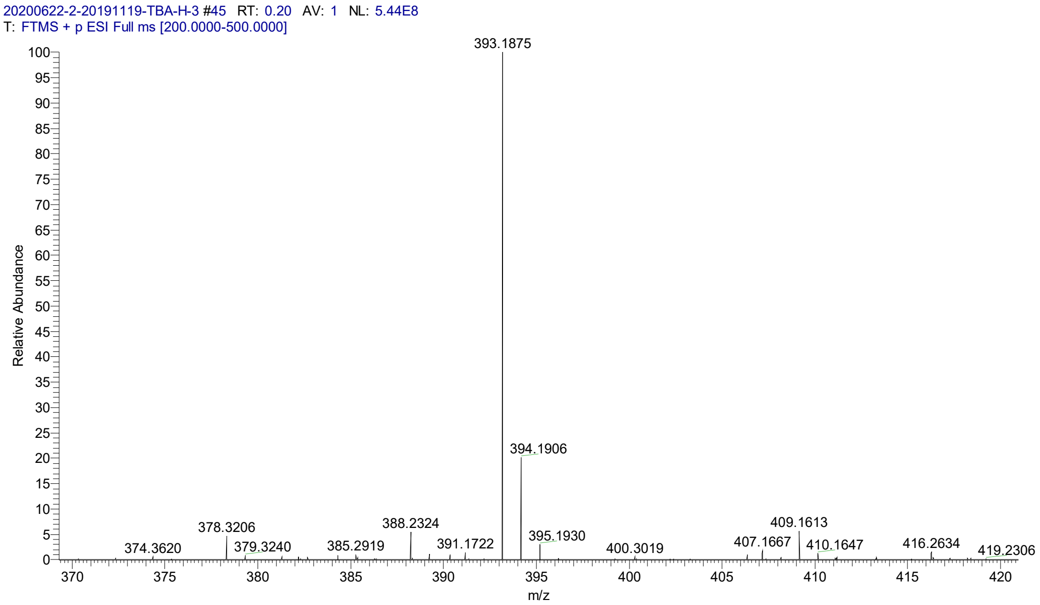

Figure S24 HRESIMS spectrum of compound **9**

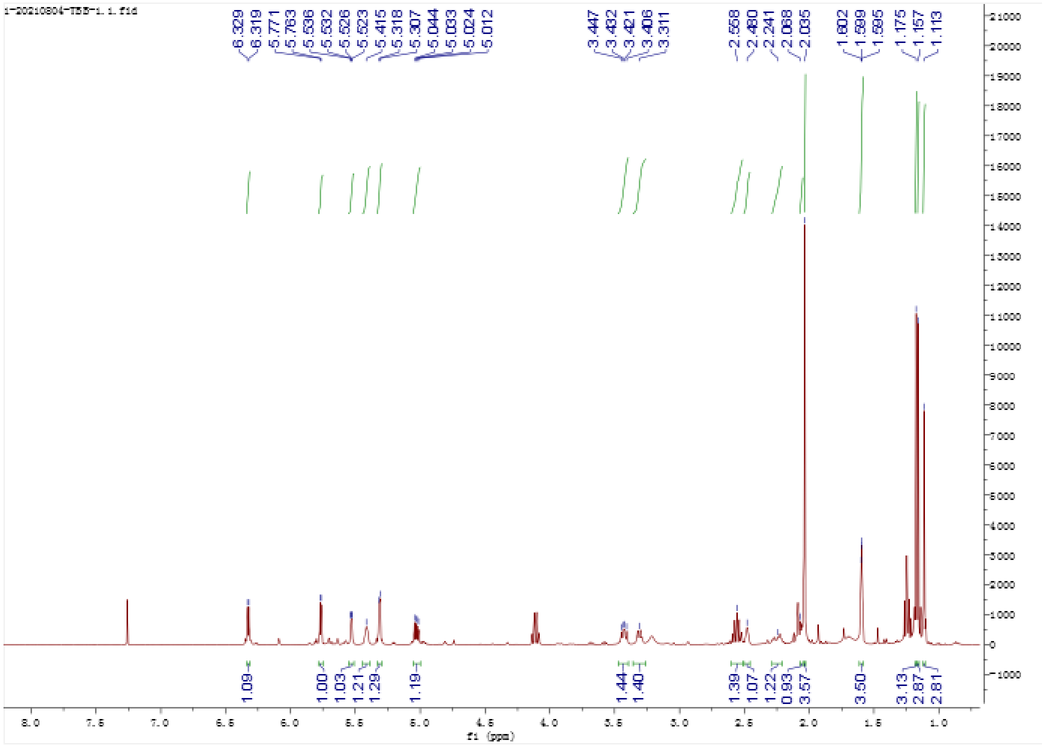

Figure S25 ^{1}H NMR spectrum of compound **10** (400 MHz in $CDCl_3$)

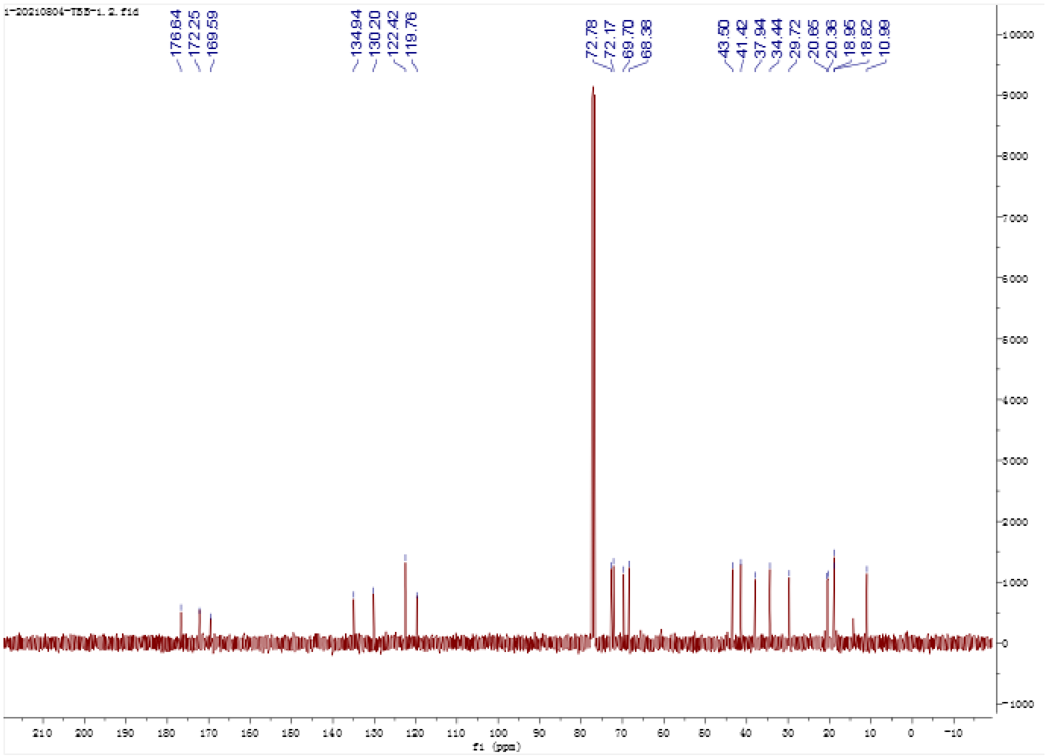

Figure S26 ^{13}C NMR spectrum of compound **10** (100 MHz in $CDCl_3$)

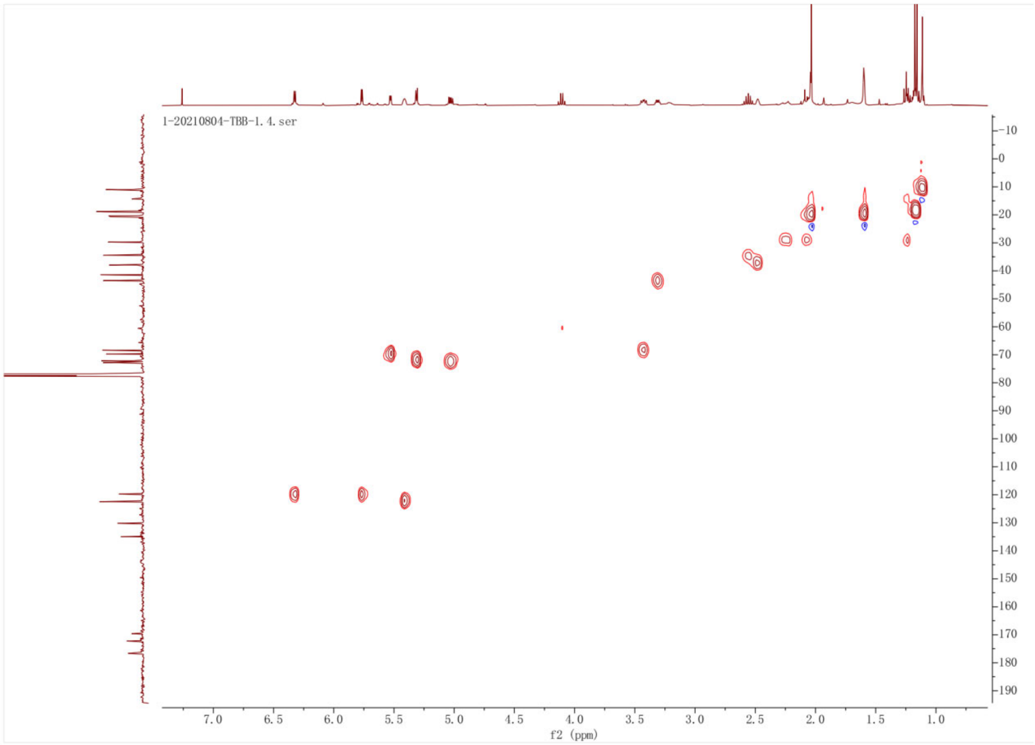

Figure S27 HSQC spectrum of compound **10** (100 MHz in $CDCl_3$)

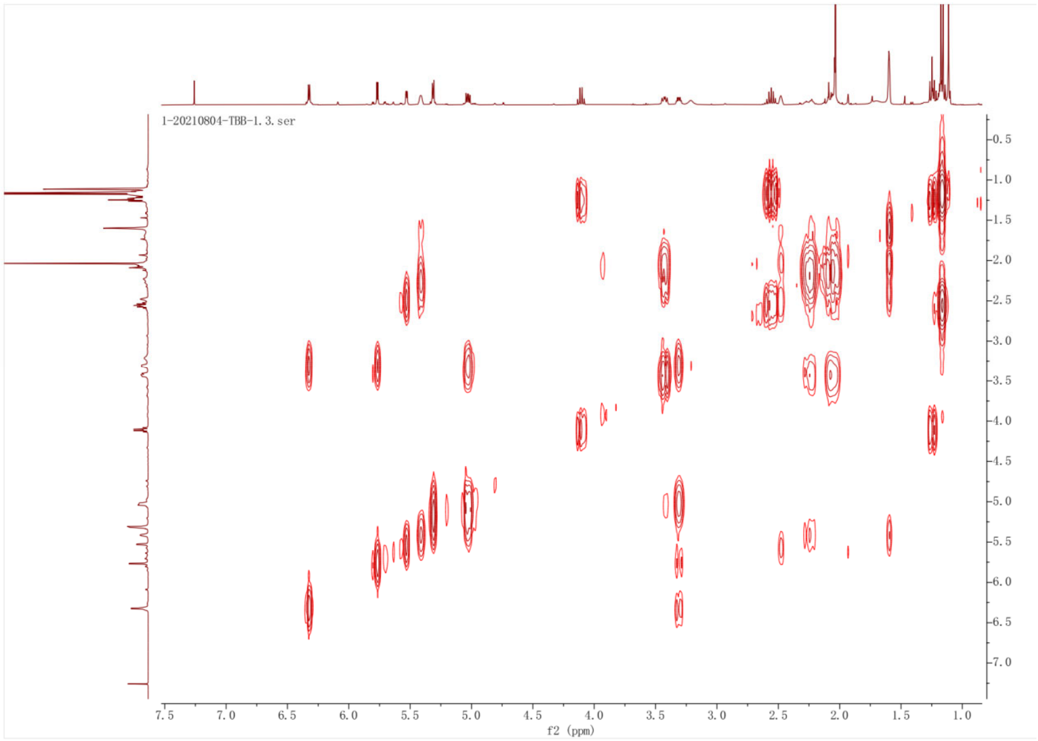

Figure S28 ^{1}H-^{1}H COSY spectrum of compound **10** (100 MHz in $CDCl_3$)

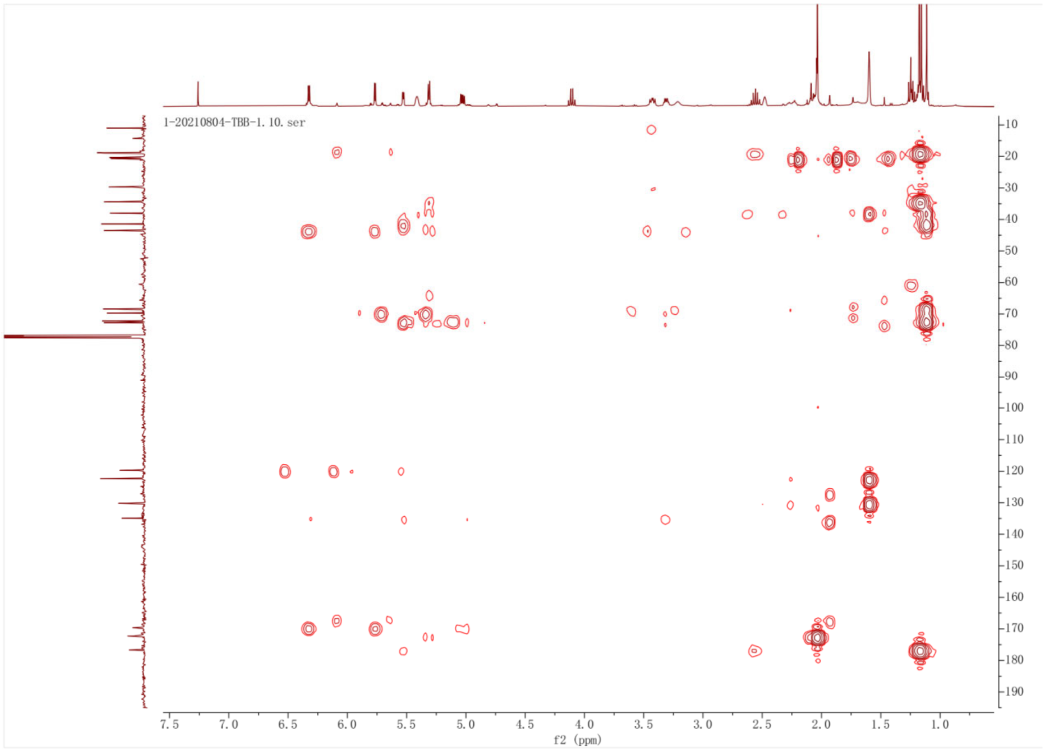

Figure S29 HMBC spectrum of compound **10** (100 MHz in $CDCl_3$)

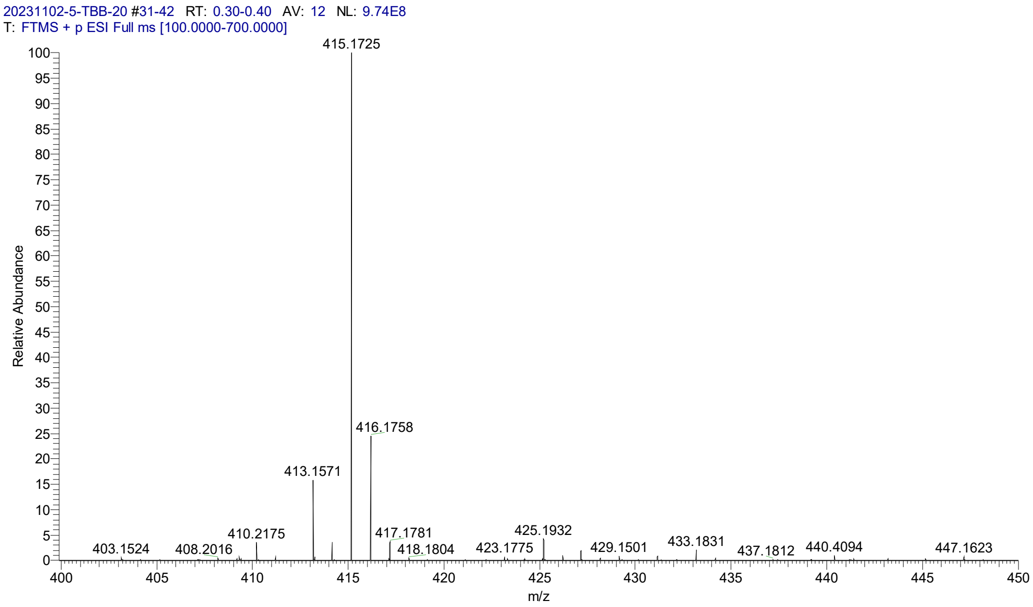

Figure S30 HRESIMS spectrum of compound **10**

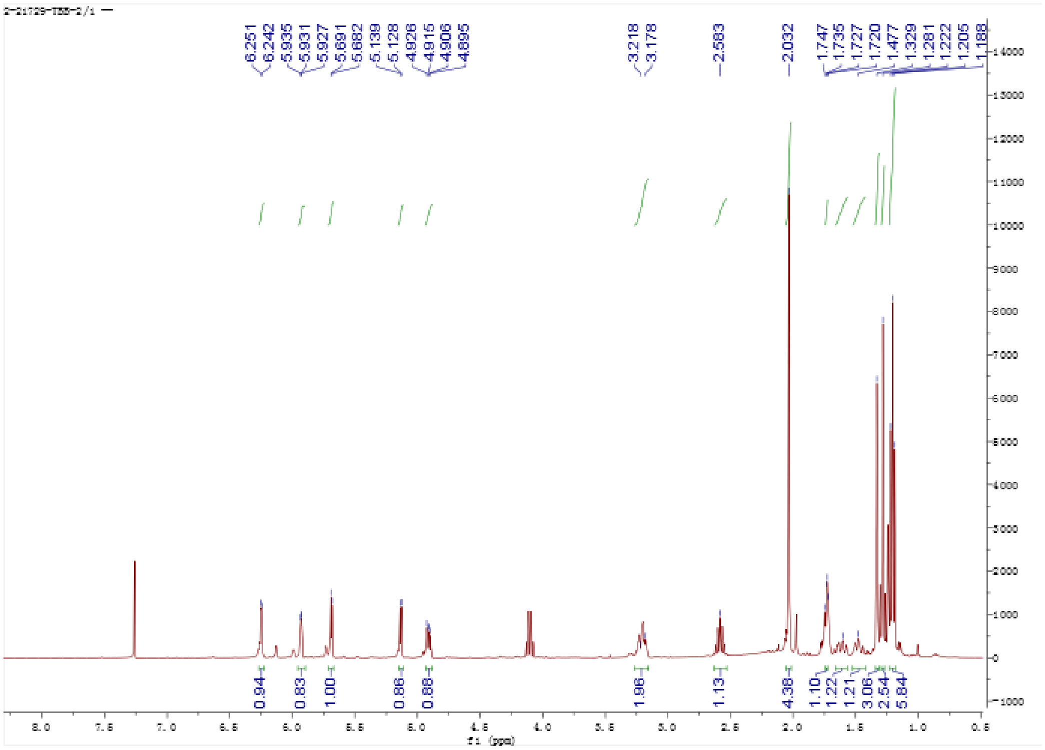

Figure S31 ^{1}H NMR spectrum of compound **11** (400 MHz in Methanol-d_4)

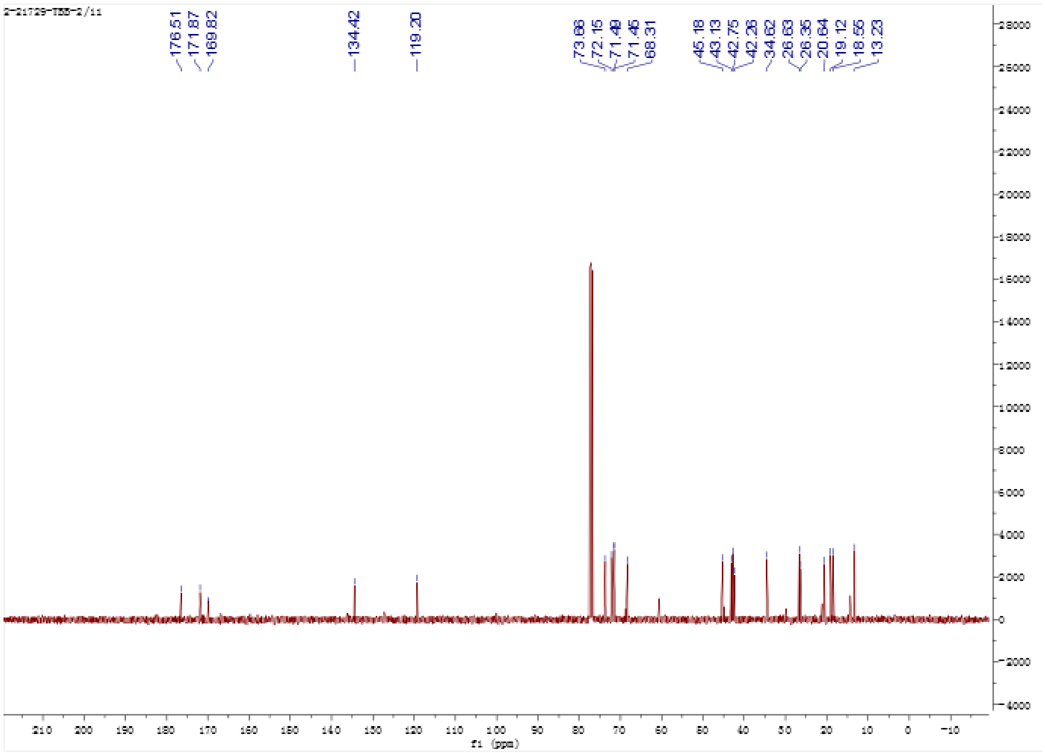

Figure S32 ^{13}C NMR spectrum of compound **11** (100 MHz in Methanol-d_4)

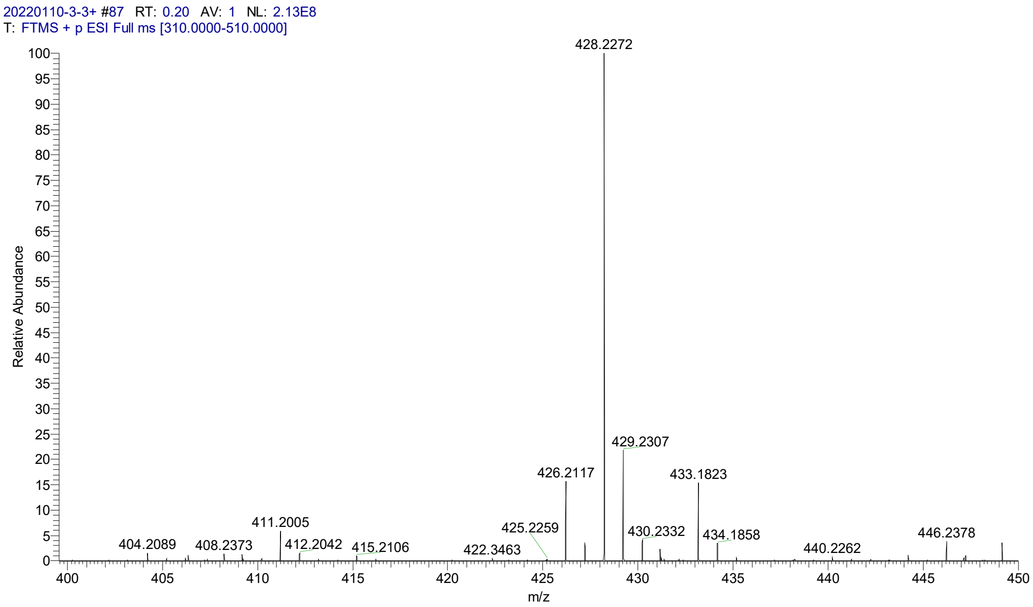

Figure S33 HRESIMS spectrum of compound **11**

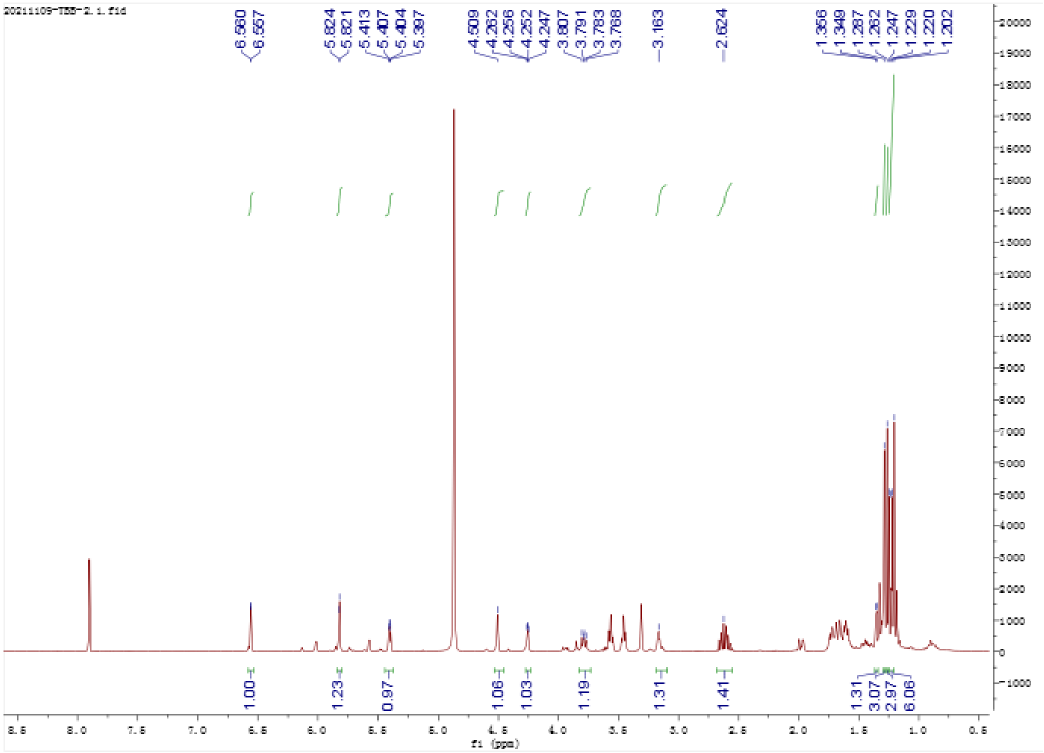

Figure S34 ¹H NMR spectrum of compound **12** (400 MHz in Methanol-d_4)

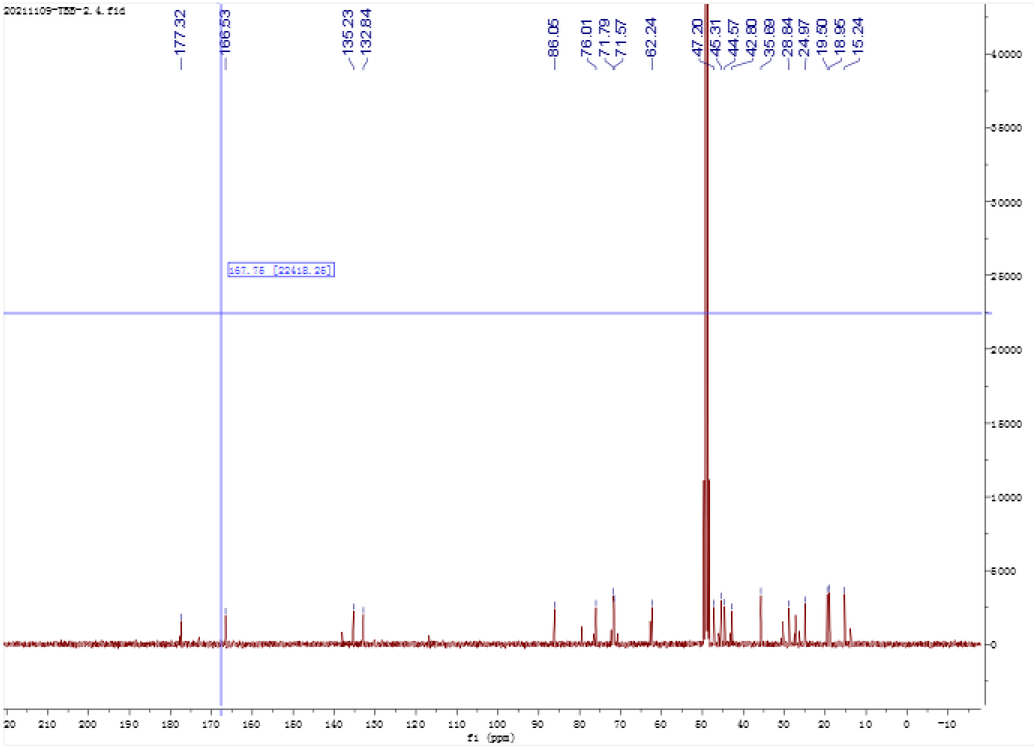

Figure S35 ¹³C NMR spectrum of compound **12** (100 MHz in Methanol-d_4)

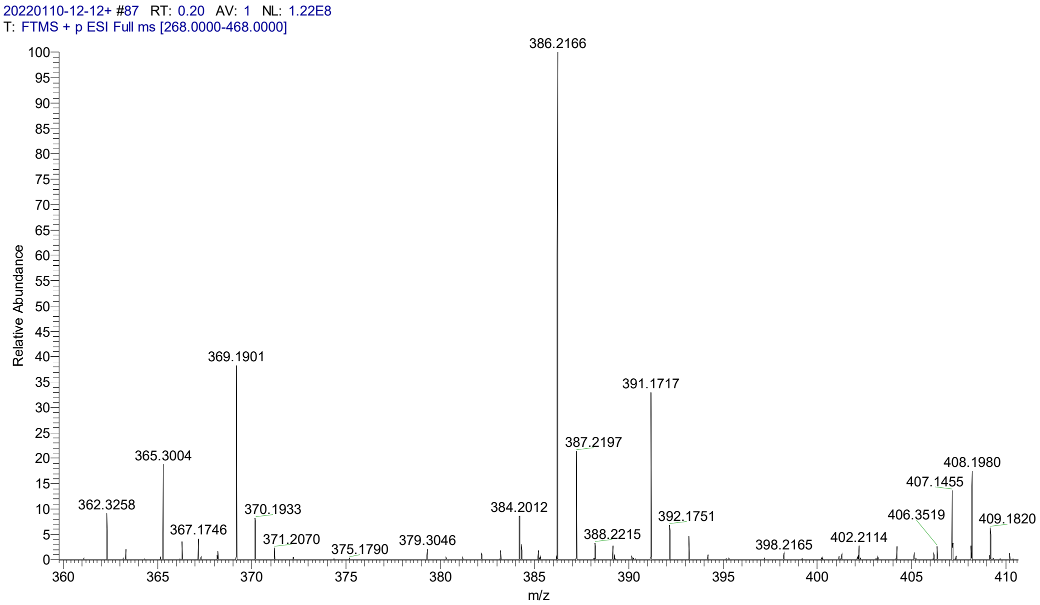

Figure S36 HRESIMS spectrum of compound **12**

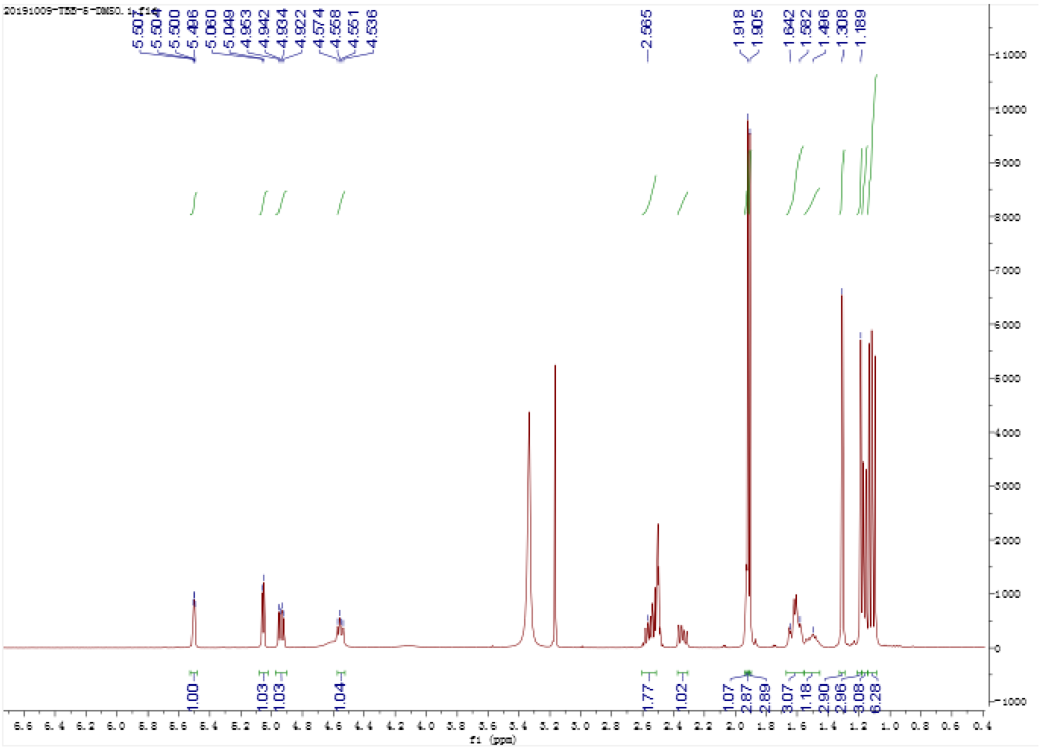

Figure S37 ^{1}H NMR spectrum of compound **13** (400 MHz in DMSO-d_6)

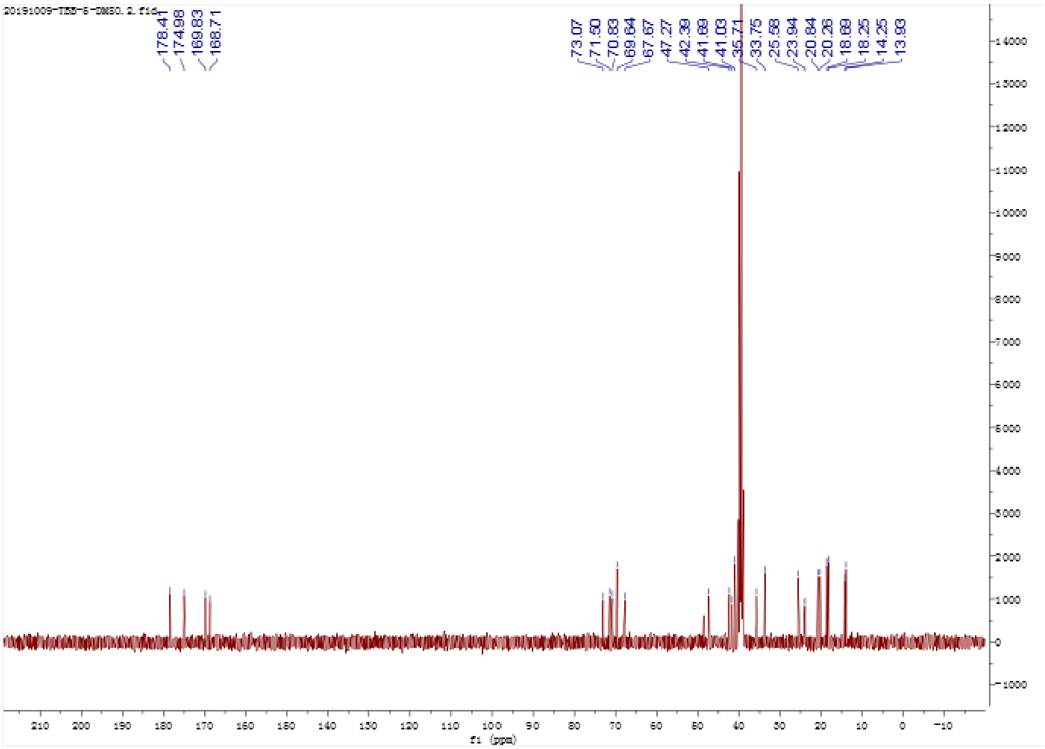

Figure S38 ^{13}C NMR spectrum of compound **13** (100 MHz in DMSO-d_6)

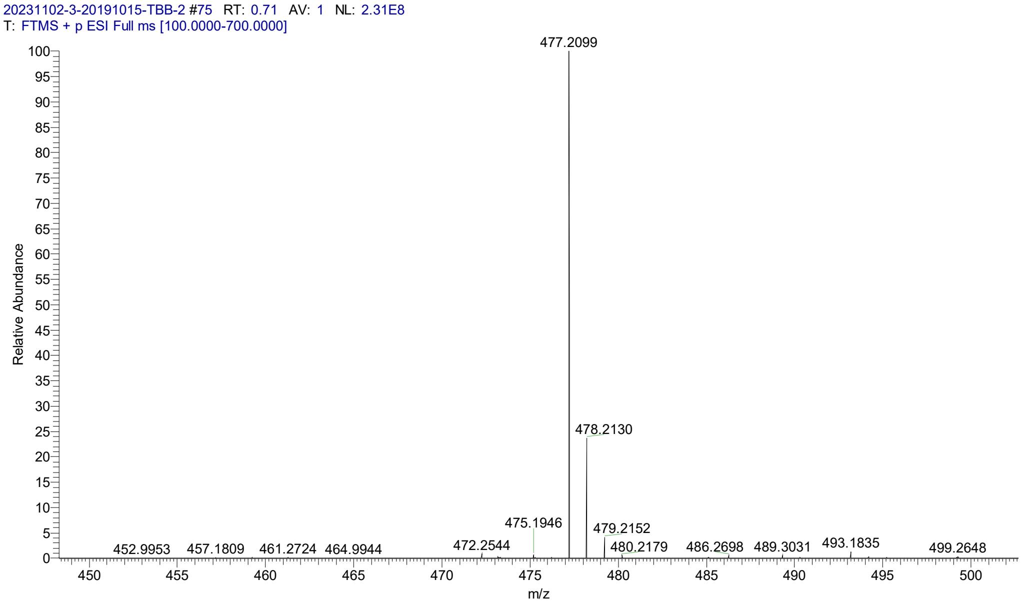

Figure S39 HRESIMS spectrum of compound **13**

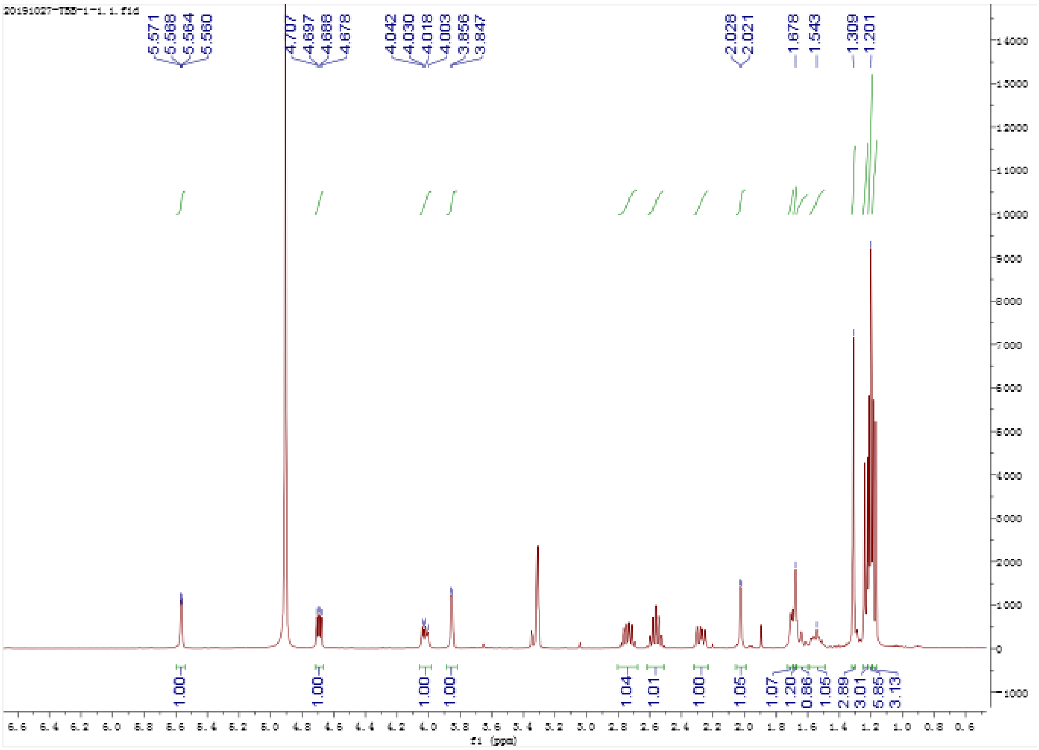

Figure S40 ^{1}H NMR spectrum of compound **14** (400 MHz in Methanol-d_4)

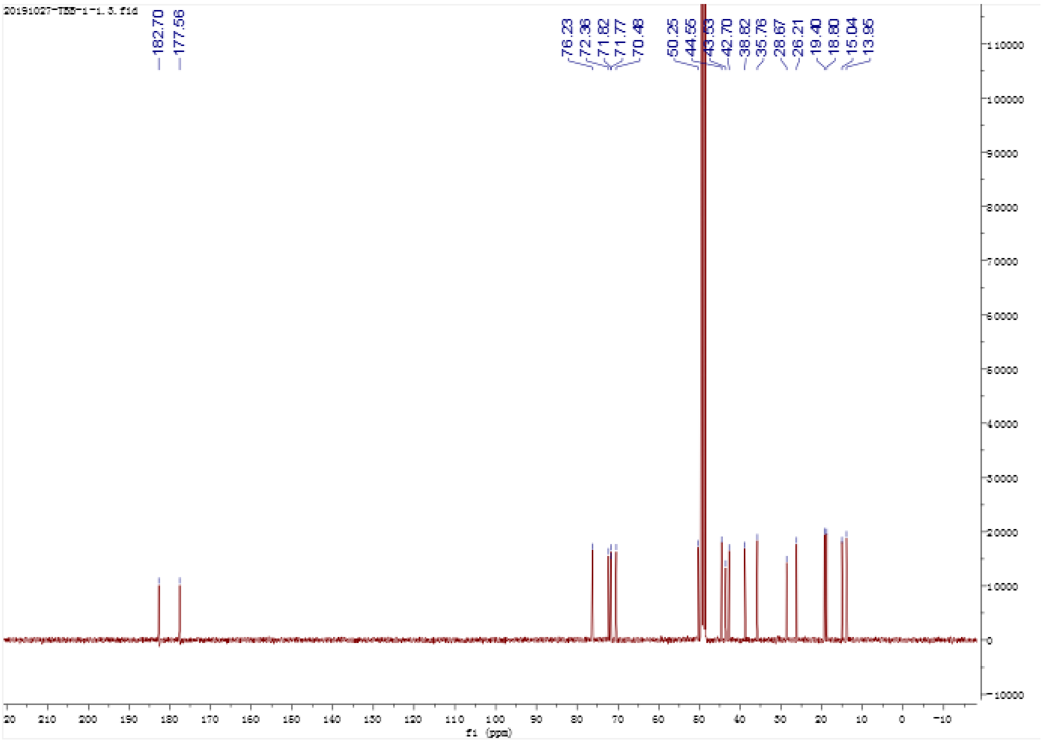

Figure S41 ^{13}C NMR spectrum of compound **14** (100 MHz in Methanol-d_4)

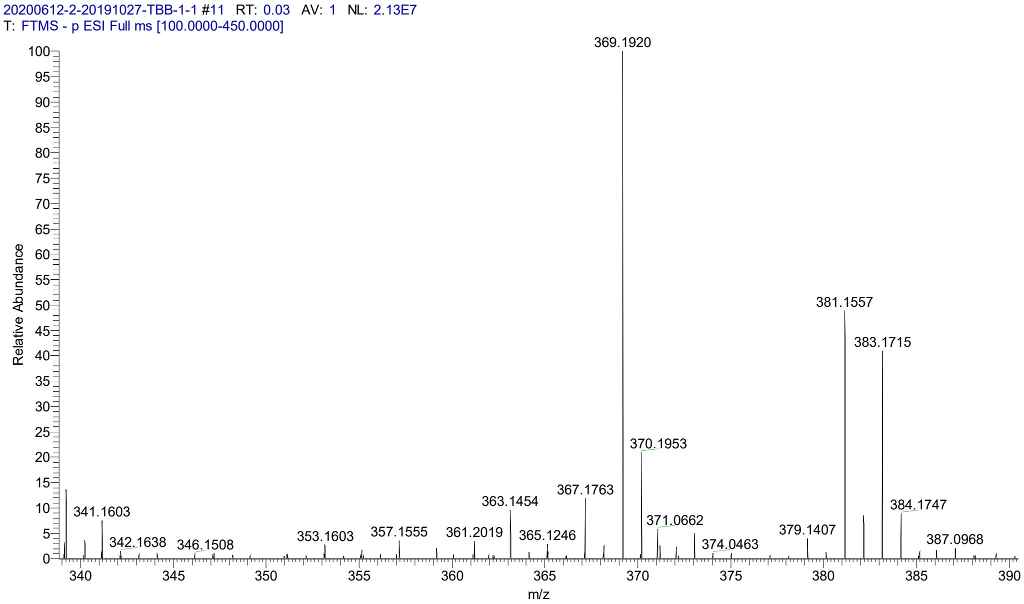

Figure S42 HRESIMS spectrum of compound **14**

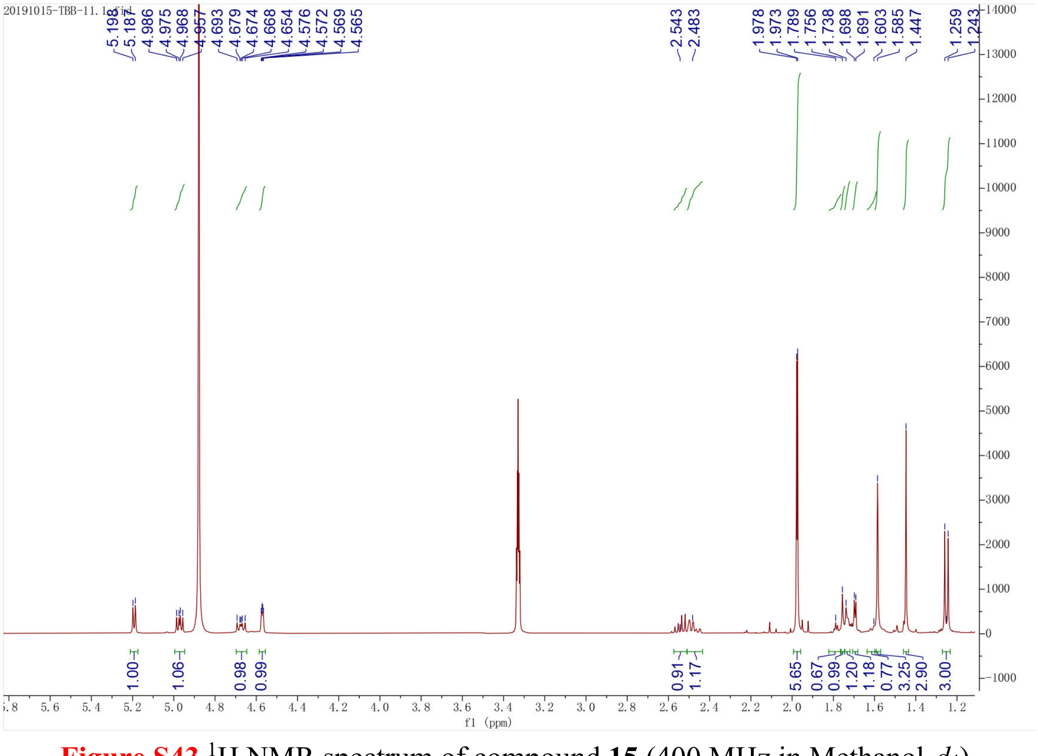

Figure S43 ^{1}H NMR spectrum of compound **15** (400 MHz in Methanol-d_4)

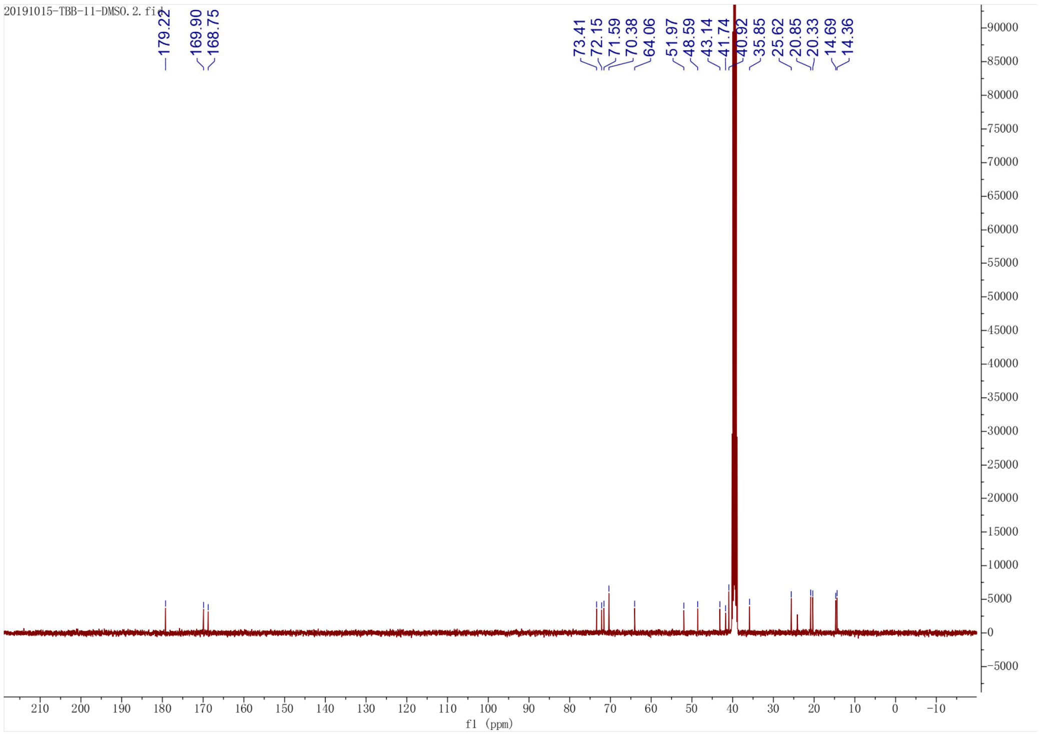

Figure S44 ^{13}C NMR spectrum of compound **15** (100 MHz in Methanol-d_4)

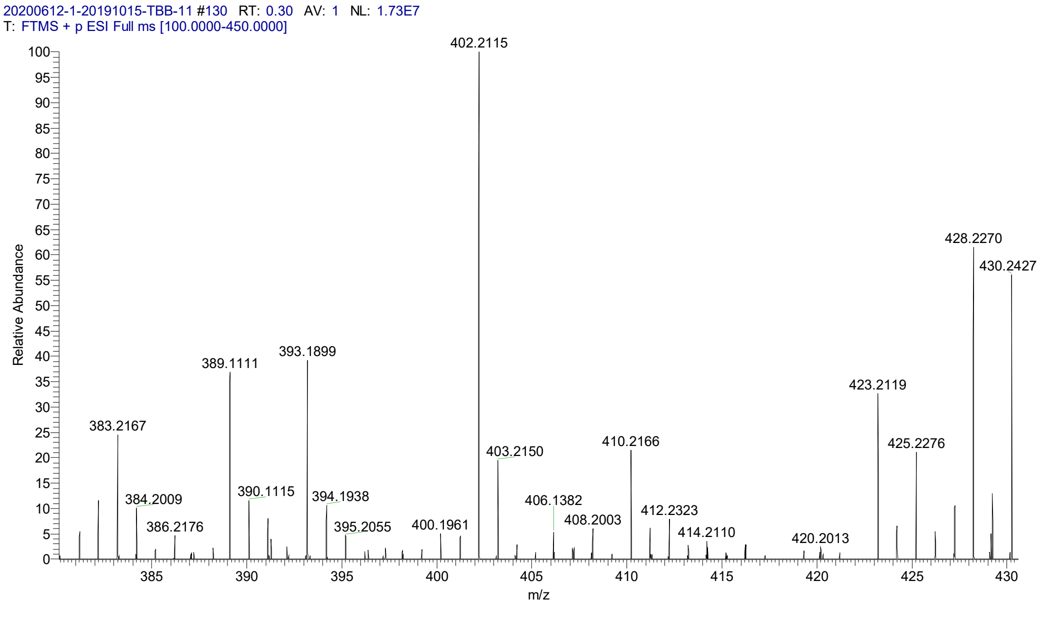

Figure S45 HRESIMS spectrum of compound **15**

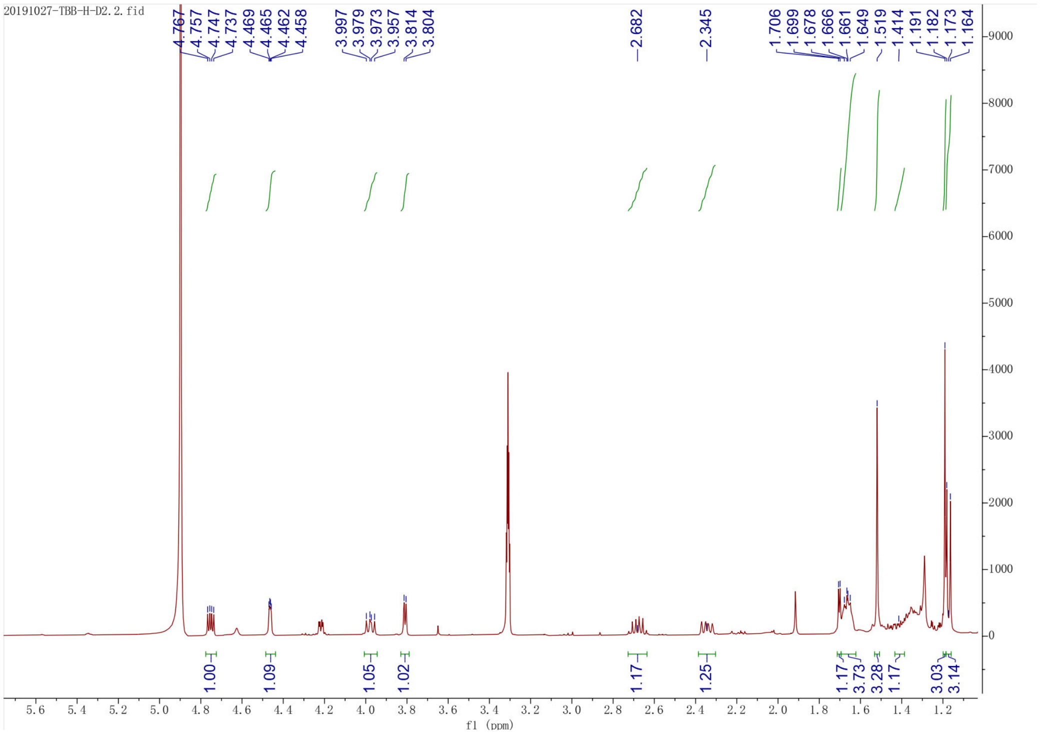

Figure S46 ^{1}H NMR spectrum of compound **16** (400 MHz inMethanol-d_4)

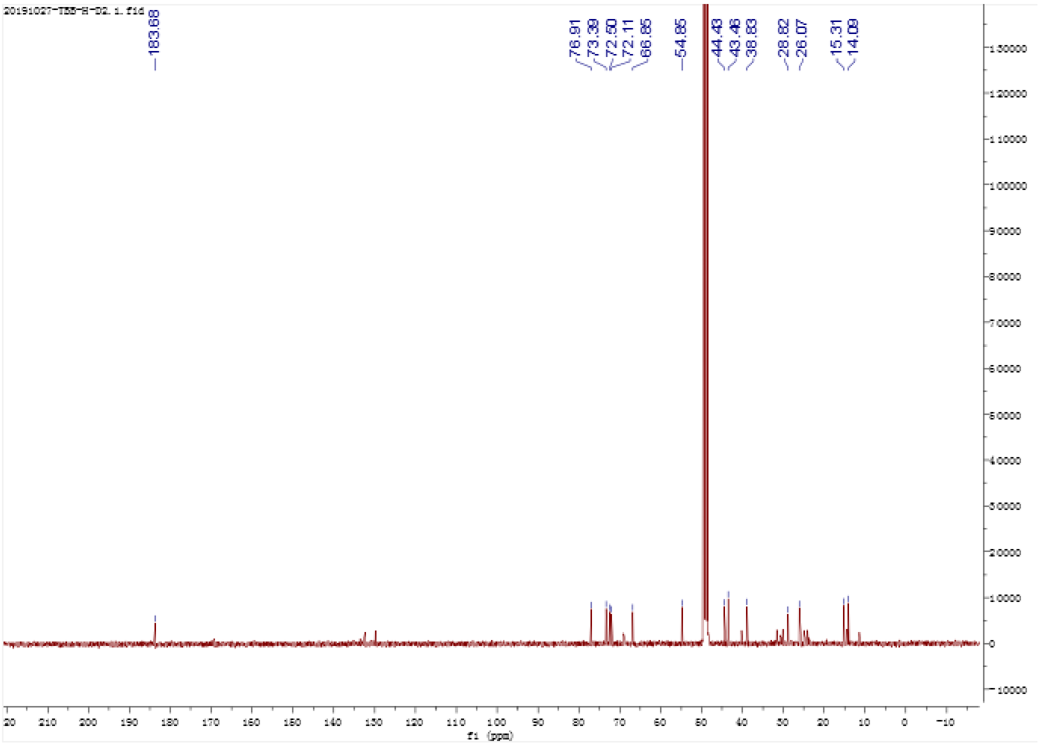

Figure S47 ^{13}C NMR spectrum of compound **16** (100 MHz in Methanol-d_4)

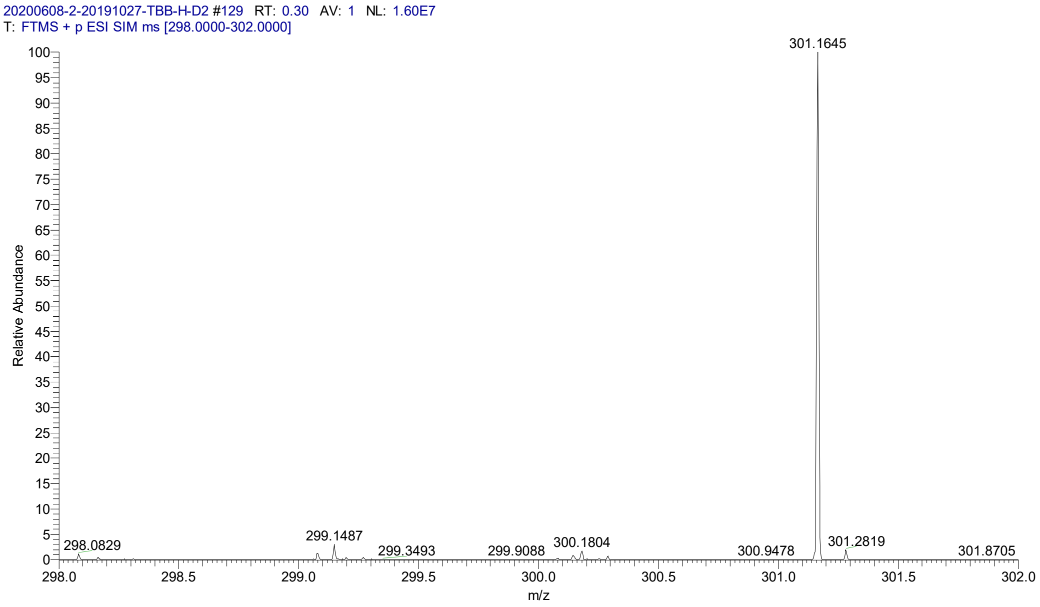

Figure S48 HRESIMS spectrum of compound **16**

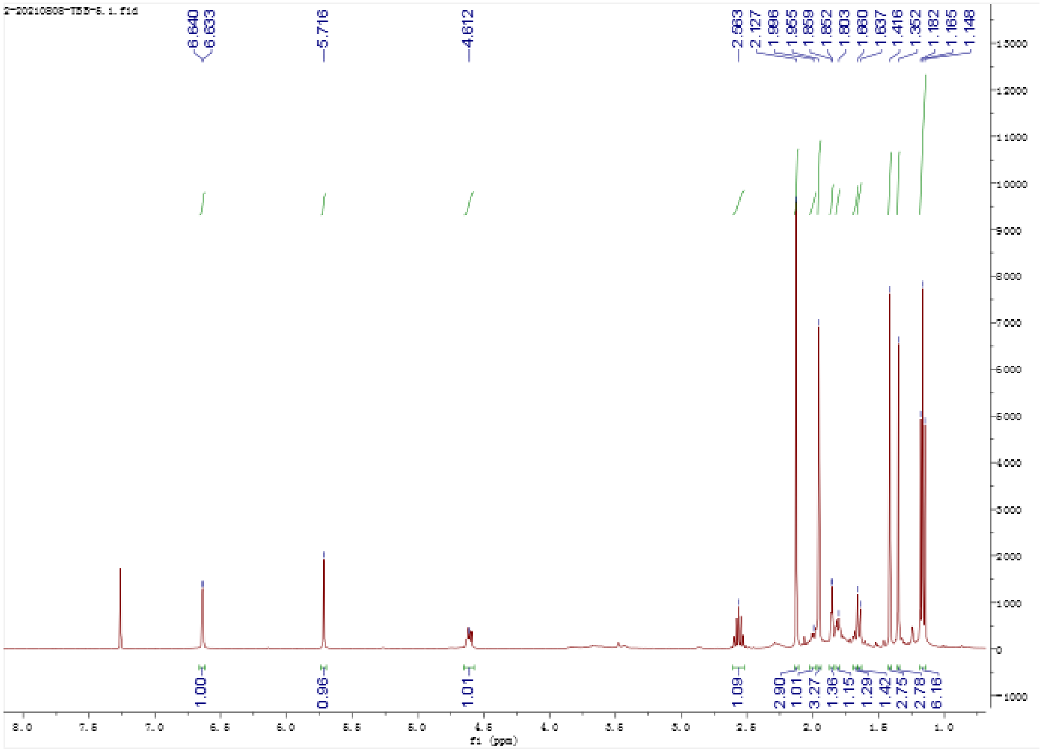

Figure S49 ^{1}H NMR spectrum of compound **17** (400 MHz in $CDCl_3$)

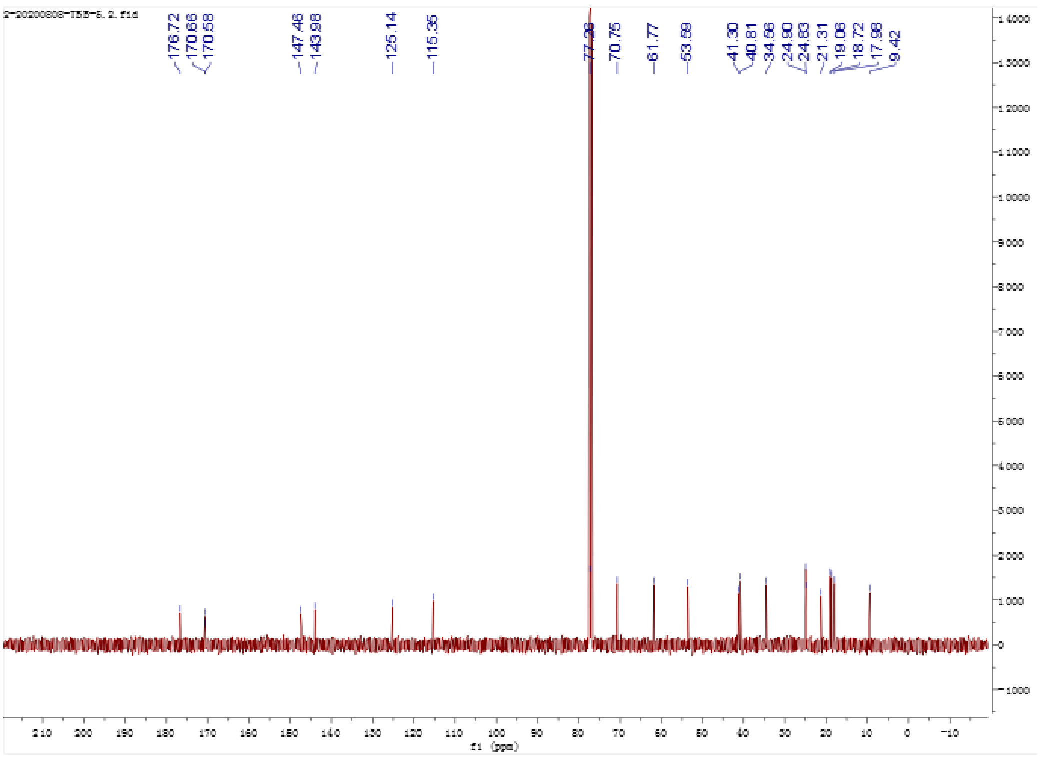

Figure S50 ^{13}C NMR spectrum of compound **17** (100 MHz in $CDCl_3$)

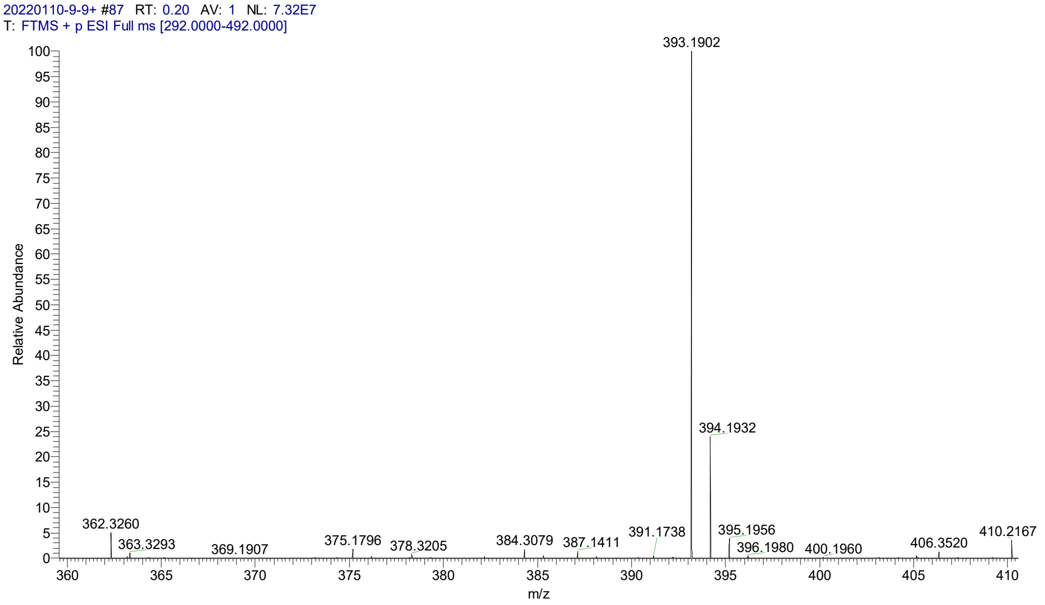

Figure S51 HRESIMS spectrum of compound **17**

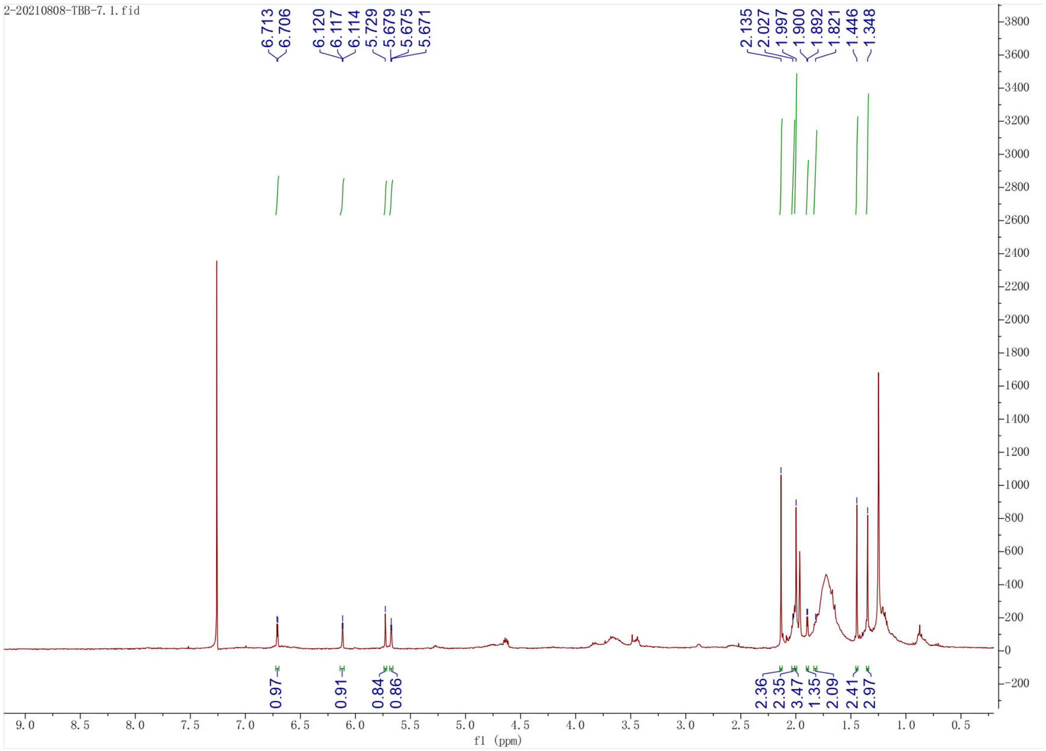

Figure S52 ^{1}H NMR spectrum of compound **18** (400 MHz in $CDCl_3$)

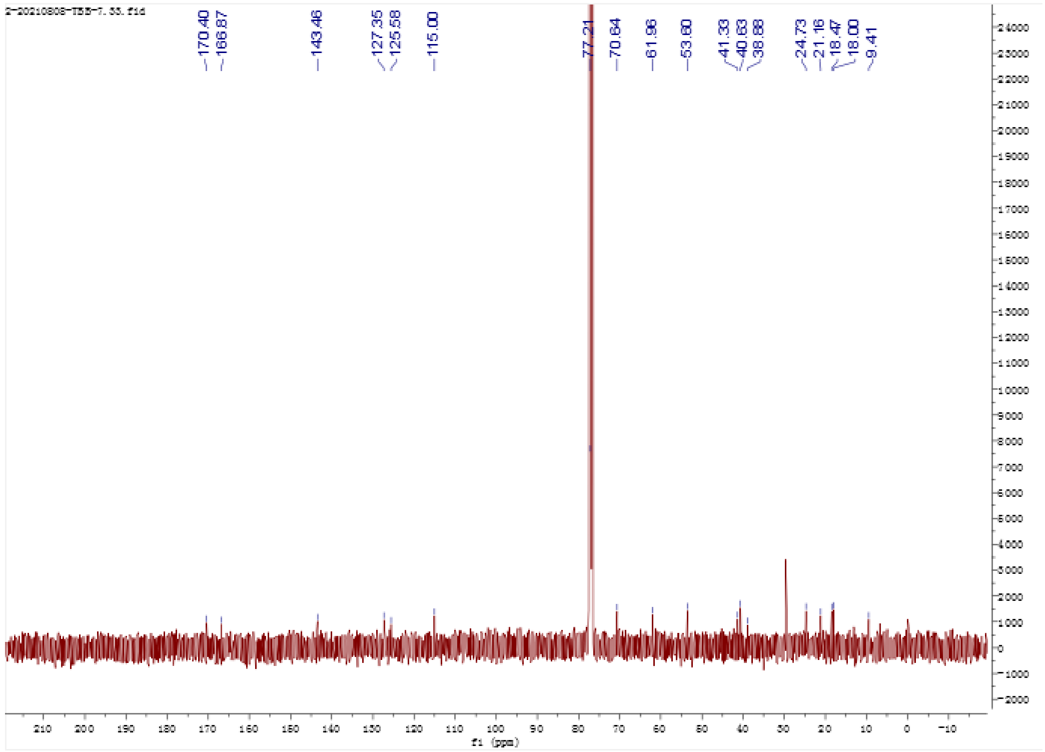

Figure S53 ^{13}C NMR spectrum of compound **18** (100 MHz in $CDCl_3$)

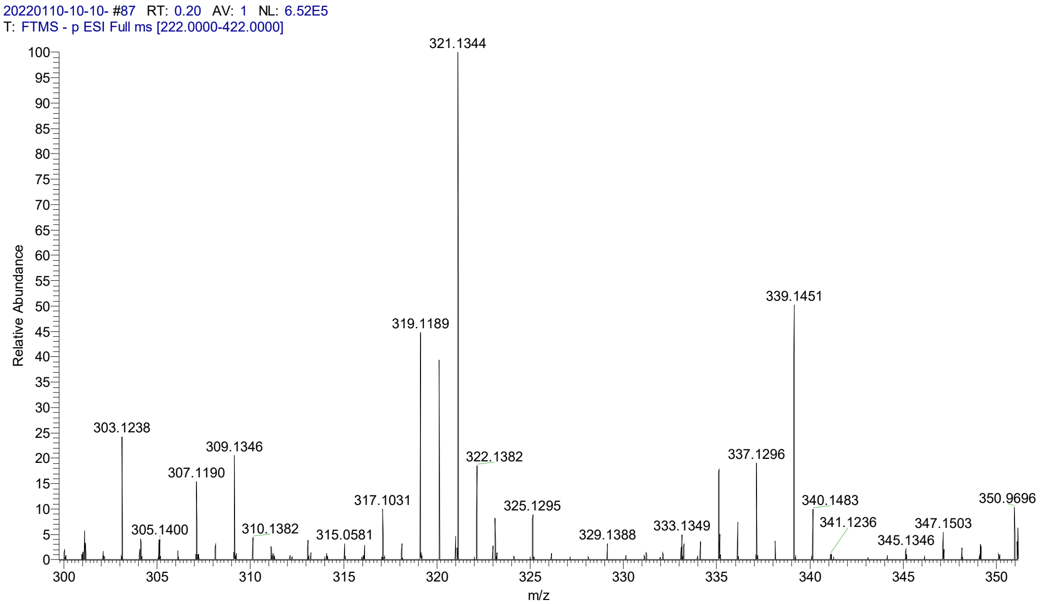

Figure S54 HRESIMS spectrum of compound **18**

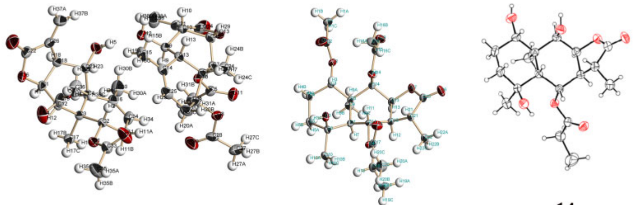

4 6 14

Figure S55. ORTEP drawing of compounds **4**, **6**, and **14**
